# Efficacy and safety of prebiotics, probiotics, and synbiotics on hemoglobin and anemia in the pediatric population: A systematic review and meta-analysis

**DOI:** 10.1371/journal.pone.0354681

**Published:** 2026-07-29

**Authors:** Gladys Estheysi Elías Solís, Steffi Aleksandra Maraza Pacori, Salomon Huancahuire-Vega, David R. Soriano-Moreno

**Affiliations:** School of Medicine, Universidad Peruana Unión, Lima, Peru; Airlangga University Faculty of Medicine: Universitas Airlangga Fakultas Kedokteran, INDONESIA

## Abstract

**Aim:**

To evaluate the efficacy and safety of prebiotics, probiotics, and synbiotics on hemoglobin levels and anemia in the pediatric population.

**Methods:**

We conducted a systematic review and meta-analysis of randomized clinical trials (RCTs). PubMed, Embase, and CENTRAL were searched up to August 2025. We included RCTs assessing prebiotics, probiotics, or synbiotics on hemoglobin or anemia in children. Random-effects meta-analyses were performed, and certainty of evidence was assessed using GRADE and minimal important differences. The protocol was registered in PROSPERO (CRD420251108276).

**Results:**

Nineteen RCTs involving 4188 children were included. Compared to placebo or standard care, prebiotics, probiotics, and synbiotics (alone or with iron) showed no important effects on hemoglobin, hematocrit, or ferritin (certainty: high to very low). However, probiotics versus placebo may reduce anemia (1 RCT; RD: -−6 per 100; 95% CI: -−15 to +6, low certainty). No serious adverse events were reported; observed events were mainly mild gastrointestinal symptoms.

**Conclusion:**

Prebiotics, probiotics, and synbiotics do not provide clinically important benefits on hemoglobin or anemia in children, although they appear safe. Larger, high-quality trials in high-risk populations are needed.

## Introduction

Anemia is a global public health problem and mainly affects the pediatric population. The World Health Organization (WHO) estimates that approximately 269 million infants aged 6–59 months are anemic [1–3]. In addition, in 2021, anemia caused 52 million years lived with disability, with dietary iron deficiency being the main cause [3]. In the pediatric population, anemia is related to poor cognitive and motor development. It can cause chronic and irreversible cognitive impairment, resulting in poor school performance and a decrease in work capacity in adulthood. It can also generate susceptibility to infections, enhancing the risk of mortality during childhood [4].

According to the WHO, the management and prevention of anemia includes iron supplementation, nutrition education, food fortification with iron compounds, and the control of parasitic and infectious diseases [5]. Early treatment is of utmost importance, as each 1 g/dL increase in hemoglobin reduces mortality by 24%, thereby preventing approximately 1.8 million deaths annually in children aged 28 days to 5 years [6]. Therefore, in recent years, other interventions have been investigated to reduce anemia in this population. Probiotics, prebiotics, and synbiotics have emerged as low-cost, accessible complementary alternatives with few side effects that have shown benefits in other outcomes in the pediatric population, such as the reduction of upper respiratory tract infections, prevention of necrotizing enterocolitis, reduction of antibiotic-associated diarrhea, reduction of pneumonia associated with mechanical ventilation, and improvement of the lipid profile [7–11].

Probiotics, defined as live microorganisms that confer benefits to host health when administered in adequate amounts [12], exert positive effects at the intestinal and systemic levels, including modulation of the immune response, increased IgA production, and competition with pathogenic bacteria [13]. For its part, prebiotics are selective fermentable substrates that induce favorable changes in the composition and activity of the intestinal microbiota [14]. Likewise, synbiotics – combinations of probiotics and prebiotics – could generate beneficial synergistic effects on health [14]. In the pediatric population, these interventions could increase hemoglobin levels by improving iron bioavailability through various mechanisms, including favoring its absorption in the duodenum and proximal colon, increasing mucin production in enterocytes, exerting immunomodulatory effects that promote an anti-inflammatory intestinal environment, and increasing the production of metabolites, such as short-chain fatty acids, that facilitate iron absorption [13,15].

A previous systematic review evaluated the effects of probiotics and prebiotics on hemoglobin levels in women of reproductive age and children, finding insufficient evidence for their effects on hemoglobin, although, it may increase ferritin levels [16]. Another systematic review evaluated the effects of milk fortified with micronutrients, probiotics, prebiotics, or synbiotics in children and found that the intervention reduced the risk of anemia. However, hemoglobin levels did not change [17]. This evidence is limited, heterogeneous, and also does not differ from the separate effects of prebiotics, probiotics, or synbiotics. In this scenario, it is necessary to develop a systematic and rigorous review that synthesizes the best available evidence on the effects of probiotics, prebiotics, and synbiotics on hemoglobin and anemia levels in the pediatric population, allowing clinical decision-making to be guided and contributing to the approach of public health policies aimed at the prevention and management of childhood anemia.

Therefore, this systematic review aimed to evaluate the efficacy and safety of prebiotics, probiotics, and synbiotics on hemoglobin levels and anemia in the pediatric population.

## Methods

### Design

The study protocol of the systematic review was registered in PROSPERO (registration number: CRD420251108276). We followed the guidelines of the Preferred Reporting Items for Systematic Review and Meta-Analysis (PRISMA) (S1 Checklist).

### Eligibility criteria

We included studies that met the following criteria: 1) design: randomized clinical trials (RCTs); 2) population: under 18 years with or without anemia; 3) intervention: prebiotics, probiotics or synbiotics, with or without supplements or fortified foods; 4) comparator: placebo, absence of intervention, iron control; and (5) that report at least one main outcome of hemoglobin and/or anemia, being considered as hematocrit, ferritin, iron absorption, and adverse events.

Case reports, opinion articles, conference abstracts, systematic reviews, narrative articles, observational studies, non-randomized trials, studies with duplicate populations, and those not available in full text were excluded.

### Literature search

Systematic searches were performed in the Cochrane Central Register of Controlled Trials (CENTRAL), Embase, and PubMed databases on August 5, 2025, with no language or publication date restrictions. Additionally, clinicaltrials.gov and Google Scholar were searched (the first 100 records were reviewed). The search was complemented by reviewing the references of the included studies and previous systematic reviews related to the subject. The complete search strategy for each database is presented in S2 Table.

### Studies selection

The elimination of duplicates and the selection process were performed in Rayyan’s software. Two reviewers independently evaluated the titles and abstracts to identify studies potentially relevant for inclusion. The full texts of the selected studies were independently assessed to determine their final eligibility. The discrepancies were resolved by consensus among the authors.

### Data extraction

The information of interest in the studies was extracted independently by the authors in a Microsoft Excel sheet. The relevant information extracted was first author’s surname, year of publication, country, the type of population studied, sample size, relevant baseline characteristics (age, sex, hemoglobin, % of anemia), details of the intervention and the comparator, financing of the study and results of the outcomes. For numerical outcomes, whenever possible, the pre-post difference calculated by the study was extracted. If the data were unavailable, the pre-post difference was calculated using the baseline and final means and their standard deviations, with a correlation of 0.5 as in one of the included studies [18]. If the study did not provide any of these data, the difference between the final values was calculated. Medians with interquartile ranges were converted to means with standard deviations. All calculations and formulas used were based on the Cochrane Manual, chapter 6 [19].

### Bias risk

The risk of bias was independently assessed by the authors using the Cochrane RoB-1 tool for RCTs. In the event of a discrepancy between the authors, a third author was consulted, who served as the settlor.

### Synthesis

For the synthesis of the evidence, the studies were grouped by intervention type, yielding six comparisons: prebiotics, prebiotics with iron, probiotics, probiotics with iron, synbiotics, and synbiotics with iron. Meta-analyses were performed when at least 2 studies were available for each comparison using RevMan Web Software. For numerical outcomes (hemoglobin, hematocrit, ferritin), mean differences (MD) with 95% confidence intervals (95% CI) were calculated. For categorical outcomes (anemia), relative risks (RR) with 95% CI were calculated. For the meta-analyses, random-effects models were used with the inverse-variance method. The Wald method was used to calculate 95% CIs when fewer than 4 studies were available, and the HKSJ method was used when 4 or more studies were available, and heterogeneity was present. Publication bias was not evaluated using statistical methods because fewer than 10 studies were included in each meta-analysis; however, there was no qualitative suspicion of this bias. No subgroups were evaluated as there was generally no heterogeneity in the results.

### Certainty of evidence

The certainty of the evidence was evaluated by outcome using the minimally contextualized GRADE approach for systematic reviews [20]. Because the studies were RCTs, they were initiated with high certainty. The imprecision was evaluated by defining the following minimum important differences (MID) [21]: ≥ 1.0 g/dL increase in hemoglobin, 5 cases per 100 absolute difference in anemia, ≥ 3% increase in hematocrit, and ≥20 ng/mL increase in ferritin. If the 95% CI of the study crossed one or two DMIs, the certainty was decreased by one or two levels, respectively. To address the risk of bias, one or two levels were reduced based on the qualitative evaluation of the studies included in each meta-analysis and their weight within it. Heterogeneity was evaluated using the I² statistic and the magnitude and direction of each study’s point estimates relative to the MDI. When studies presented discrepant point estimates relative to each other or to the MDI, the certainty of the evidence was reduced by 1 or 2 levels [21]. Publication bias was evaluated qualitatively, taking into account the potential for bias in small studies with positive or striking results and in those funded by industry. No indirect evidence was considered, as the PICO questions were similar. The findings were presented using summary of findings (SoF) tables and communicated using the GRADE informative statements [20].

## Results

### Studies selection

Initially, we found 625 articles in the database search. After removing duplicates, we evaluated 492 articles by title and abstract, of which 65 were revised to full text. Finally, 19 RCTs were included [22–40] (S3 Fig). The exclusion reasons for the articles evaluated in full text are found in S4 Table.

### Characteristics of the included studies

The included RCTs total 4188 participants, ranging from 24 to 781. The studies were conducted in various countries in Asia, Europe, Africa, and Oceania, including Pakistan [22], Poland [24], China [31], Brazil [25,28,32], Kenya [27,35,36], Indonesia [33,38–40], India [23,30], Egypt [34], Finland [37], Vietnam, [29] and New Zealand and Australia [26]. The studies included infants to adolescents, and some focused on specific populations such as children with severe acute malnutrition [22], moderate to severe protein-energy malnutrition [23], children with anemia or iron deficiency [30,33,39,40], children with celiac disease [24], and infants at high risk of allergies, along with their mothers [37]. Regarding baseline characteristics, the mean hemoglobin ranged from 8.7 to 13.2 g/dL, the average age ranged from 0.5 to 11.1 years, and the proportion of men ranged from 0% to 63.5%. In addition, the prevalence of anemia ranged from 16.6% to 100%, although not all studies reported it. The interventions evaluated included prebiotics [22,24,25,28,31], prebiotics combined with iron [27,35,36,39], probiotics [23,34,38], probiotics combined with iron [32,40], synbiotics [29,30,37], and synbiotics combined with iron [26,33]. The most frequently evaluated prebiotics were galacto-oligosaccharides, followed by fructo-oligosaccharides and inulin, while the most studied probiotics corresponded to strains of the genus Lactobacillus. The comparators consisted of a placebo and a standard control [22–26,28–31,34,37,38] or standard control with iron [27,32,33,35,36,39,40] (S5 Table). The details of the intervention, the comparator, and funding in each study are found in S6 Table.

### Risk of bias

Most studies were at high risk of bias due to incomplete outcome data. In addition, a significant proportion of studies showed unclear bias in the selective reporting of results and other biases due to unbalanced baseline characteristics. On the other hand, all studies had a low risk of outcome blinding bias. Specifically, the RCTs with the greatest bias were Kuitunen – 2009, Mohammad – 2006, Silva – 2008, and Xuan – 2013 (S7 Fig).

### Outcomes

The results of the studies are summarized in the SoF Tables (Tables 1–6). The adverse events detailed for each study are in S8 Table. Forest plots are found in S9 Fig.

**Table 1:** Summary of results of the effects of prebiotics versus placebo in the pediatric population.

Population	Pediatric patients (<18 years)						
Intervention	Prebiotics						
Comparator	Placebo						
Outcomes	Number of patients (number of studies)	Intervention: prebiotics	Comparator: placebo	Relative effect (95% CI)	Absolute effect (95% CI)	Certitude	Interpretation
Increase in hemoglobin (g/dL)	787 (5 RCTs)	Average range: −0.23 to +0.8	Average range: −0.09 to +0.26	–	MD: + 0.15 (−0.37 to +0.67)	⨁⨁⨁⨁ High	Compared to a placebo, prebiotics do not produce an important effect on the increase in hemoglobin
Presence of anemia	No studies						
Increase in hematocrit (%)	747 (4 RCTs)	Average range: −0.4 to +8.96	Average range: −0.69 to +1.1	–	MD: −0.04 (−0.44 to +0.36)	⨁⨁⨁◯^a^ Moderate	Compared to a placebo, prebiotics probably do not produce an important effect on increasing hematocrit
Increase in ferritin (ng/ml)	543 (3 RCTs)	Average range: −2.84 to +1.67	Average range: −4.54 to −0.33	–	MD: + 2.24 (+0.77 to +3.70)	⨁⨁⨁⨁ High	Compared with placebo, prebiotics do not importantly increase ferritin levels.
Adverse events	The majority of RCTs reported no serious adverse events (Batool – 2023; Feruś – 2018). The observed events were mainly gastrointestinal, mild, and similar across groups (Maximino – 2024). In isolation, one case of food allergy (Li – 2014) and a higher frequency of oral candidiasis were reported in the intervention group (Pontes – 2016).						

RCT: Randomized Clinical Trial; HR: Hazard Ratio; CI: Confidence Interval; DR: Risk Difference; MD: Mean Difference

A MID of ≥1.0 g/dL was established for the increase in hemoglobin, 5 cases per 100 absolute difference for anemia, ≥ 3% for the increase in hematocrit, and ≥20 ng/ml for the increase in ferritin.

Explanations about the certainty of the evidence:

a. The certainty was reduced by one level due to inconsistency, since the point effect between the studies was heterogeneous with respect to the MDI.

**Table 2:** Summary of the effects of prebiotics plus iron versus standard control plus iron in the pediatric population.

Population	Pediatric patients (<18 years)						
Intervention	Prebiotics plus iron						
Comparator	Standard control plus iron						
Outcomes (follow-up)	Number of patients (number of studies)	Intervention: prebiotics plus iron	Comparator: Control plus iron	Relative effect (95% CI)	Absolute effect (95% CI)	Certitude	Interpretation
Increase in hemoglobin (g/dL)	382 (4 RCTs)	Average range: −0.1 to +3.28	Average range: +0.1 to +2.29	–	MD: −0.15 (−1.39 to +1.10)	⨁⨁⨁◯^a^ Moderate	Compared to the iron control, prebiotics with iron probably do not produce an important effect on the increase in hemoglobin
Presence of anemia	158 (2 RCTs)	24/78 (30.8%)	23/80 (28.8%)	RR: 1.08 (0.68 to 1.73)	DR: + 2 per 100 (−9 to +21)	⨁⨁◯◯^b^ Low	Compared to the iron control, prebiotics with iron may not produce an important effect on anemia.
Increase in hematocrit (%)	No studies						
Increase in ferritin (ng/ml)	147 (2 RCTs)	Average range: +14.68 to +18.79	Average range: +7.7 to +8.66	–	MD: −3.72 (−8.55 to +1.11)	⨁⨁⨁⨁ High	Compared to the iron control, iron prebiotics do not importantly increase ferritin levels.
Adverse events	One study noted that DOGS mitigated the adverse effects of iron – including microbiome alterations, intestinal damage, respiratory infections, inflammation, and diarrhea – without quantifying the results (Paganini – 2017b). Likewise, the scGOS/lcFOS combination showed a protective effect on the microbiota against iron (Mikulić – 2019).						

RCT: Randomized Clinical Trial; HR: Hazard Ratio; CI: Confidence Interval; DR: Risk Difference; MD: Mean Difference

A MID of ≥1.0 g/dL was established for the increase in hemoglobin, 5 cases per 100 absolute difference for anemia, ≥ 3% for the increase in hematocrit, and ≥20 ng/ml for the increase in ferritin.

Explanations about the certainty of the evidence:

a. One level reduced the certainty due to inaccuracy, since the 95% CI crosses a MID.

b. Two levels reduced the certainty due to inaccuracy, since the 95% CI crosses two MIDs.

**Table 3:** Summary of results of the effects of probiotics versus placebo in the pediatric population.

Population	Pediatric patients (<18 years)						
Intervention	Probiotics						
Comparator	Placebo						
Outcomes (follow-up)	Number of patients (number of studies)	Intervention: probiotics	Comparator: placebo	Relative effect (95% CI)	Absolute effect (95% CI)	Certitude	Interpretation
Increase in hemoglobin (g/dL)	581 (3 RCTs)	Average range: +0.3 to +1.14	Average range: −1.7 to 0.52	–	MD: + 0.17 (−1.42 to +1.75)	⨁◯◯◯^a,b^ Very low	Compared to a placebo, probiotics may not produce an important effect on the increase in hemoglobin, although the evidence is very uncertain
Presence of anemia	294 (1 RCT)	60/144 (41.7%)	72/150 (48.0%)	RR: 0.87 (0.68 to 1.12)	DR: −6 per 100 (−15 to +6)	⨁⨁◯◯^b^ low	Compared with a placebo, probiotics could importantly reduce anemia.
Increase in hematocrit (%)	489 (1 RCT)	Mean: −0.01	Mean: −0.01	–	MD: 0.00 (−0.05 to +0.05)	⨁⨁⨁⨁ High	Compared to a placebo, probiotics do not produce an important effect on increasing hematocrit
Increase in ferritin (ng/ml)	497 (2 RCTs)	Average range: −144.69 to −3.87	Average range: −102.17 to −4.16	–	MD: + 0.16 (−6.76 to +7.09)	⨁⨁⨁⨁ High	Compared to placebo, probiotics do not importantly increase ferritin levels.
Adverse events	Most studies reported no adverse events related to the intervention (Agustina – 2013; Mohammad – 2006). In one trial, two deaths due to bronchopneumonia were recorded, one in each group, which were not attributed to the intervention (Dewan – 2007).						

RCT: Randomized Clinical Trial; HR: Hazard Ratio; CI: Confidence Interval; DR: Risk Difference; MD: Mean Difference

A MID of ≥1.0 g/dL was established for the increase in hemoglobin, 5 cases per 100 absolute difference for anemia, ≥ 3% for the increase in hematocrit, and ≥20 ng/ml for the increase in ferritin.

Explanations about the certainty of the evidence:

a. Two levels reduced the certainty due to risk of bias, since most of the weight of the meta-analysis is composed of studies with risk of bias.

b. Two levels reduced the certainty due to inaccuracy, since the 95% CI crosses two MIDs.

**Table 4:** Summary of the effects of probiotics plus iron versus standard control plus iron in the pediatric population.

Population	Pediatric patients (<18 years)						
Intervention	Probiotics plus iron						
Comparator	Standard control plus iron						
Outcomes (follow-up)	Number of patients (number of studies)	Intervention: probiotics plus iron	Comparator: Control plus iron	Relative effect (95% CI)	Absolute effect (95% CI)	Certitude	Interpretation
Increase in hemoglobin (g/dL)	175 (2 RCTs)	Average range: −0.5 to +1.16	Average range: −0.7 to +0.98	–	MD: + 0.20 (−0.07 to +0.47)	⨁⨁◯◯^a^ low	Compared to the iron control, iron probiotics may not produce an important effect on increasing hemoglobin
Presence of anemia	No studies						
Increase in hematocrit (%)	175 (2 RCTs)	Average range: 1.5 to 1.81	Average range: −2.2 to +1.49	–	MD: + 0.52 (−0.17 to +1.20)	⨁⨁◯◯^a^ low	Compared to the iron control, iron probiotics may not produce an important effect on increasing hematocrit
Increase in ferritin (ng/ml)	109 (1 RCT)	Mean: −6.8	Mean: +5	–	MD: −11.80 (−20.29 to −3.31)	⨁◯◯◯^a,b^ Very low	Compared with the iron control, iron probiotics may not importantly increase ferritin levels, although the evidence is very uncertain.
Adverse events	None of the included studies reported information on adverse events.						

RCT: Randomized Clinical Trial; HR: Hazard Ratio; CI: Confidence Interval; DR: Risk Difference; MD: Mean Difference

A MID of ≥1.0 g/dL was established for the increase in hemoglobin, 5 cases per 100 absolute difference for anemia, ≥ 3% for the increase in hematocrit, and ≥20 ng/ml for the increase in ferritin.

Explanations about the certainty of the evidence:

a. Two levels reduced the certainty due to risk of bias, since most of the weight of the meta-analysis is composed of studies with risk of bias.

b. One level reduced the certainty due to inaccuracy, since the 95% CI crosses a MID.

**Table 5:** Summary of results of the effects of synbiotics versus placebo in the pediatric population.

Population	Pediatric patients (<18 years)						
Intervention	Synbiotics						
Comparator	Placebo						
Outcomes (follow-up)	Number of patients (number of studies)	Intervention: synbiotics	Comparator: placebo	Relative effect (95% CI)	Absolute effect (95% CI)	Certitude	Interpretation
Increase in hemoglobin (g/dL)	1307 (2 RCTs)	Mean: + 1.63	Mean: + 1.73	–	MD: −0.10 (−0.32 to +0.12)	⨁⨁⨁⨁ High	Compared to a placebo, synbiotics do not produce an important effect on increasing hemoglobin
Presence of anemia	920 (2 RCTs)	176/443 (39.7%)	194/477 (40.6%)	RR: 0.94 (0.82 to 1.08)	DR: −2 per 100 (−7 to +3)	⨁⨁⨁◯^a^ Moderate	Compared to a placebo, synbiotics probably do not produce an important effect on anemia
Increase in hematocrit (%)	1126 (2 RCTs)	Mean: + 4.85	Mean: + 4.60	–	MD: −0.04 (−0.34 to +0.27)	⨁⨁⨁⨁ High	Compared to a placebo, synbiotics do not produce an important effect on increasing hematocrit
Increase in ferritin (ng/ml)	443 (1 RCT)	Mean: + 4.30	Mean: + 4.57	–	MD: −0.27 (−2.10 to +1.56)	⨁⨁⨁⨁ High	Compared to placebo, synbiotics do not importantly increase ferritin levels.
Adverse events	One study reported no adverse events during one year of follow-up (Sazawal – 2010). In another, subclinical intestinal inflammation was evidenced by fecal biomarkers (Kuitunen – 2009), and a third study reported predominantly gastrointestinal adverse events without specifying their frequency or severity (Xuan – 2013).						

RCT: Randomized Clinical Trial; HR: Hazard Ratio; CI: Confidence Interval; DR: Risk Difference; MD: Mean Difference

A MID of ≥1.0 g/dL was established for the increase in hemoglobin, 5 cases per 100 absolute difference for anemia, ≥ 3% for the increase in hematocrit, and ≥20 ng/ml for the increase in ferritin.

Explanations about the certainty of the evidence:

a. One level reduced the certainty due to inaccuracy, since the 95% CI crosses a MID.

**Table 6:** Summary of the effects of synbiotics plus iron versus standard control with or without iron in the pediatric population.

Population	Pediatric patients (<18 years)						
Intervention	Synbiotics plus iron						
Comparator	Standard control with or without iron*						
Outcomes (follow-up)	Number of patients (number of studies)	Intervention: synbiotics plus iron	Comparator: standard control	Relative effect (95% CI)	Absolute effect (95% CI)	Certitude	Interpretation
Increase in hemoglobin (g/dL)	186 (2 RCTs)	Average range: +0.59 to +0.60	Average range: +0.24 to +0.60	–	MD: + 0.23 (−0.09 to +0.56)	⨁◯◯◯^a,b^ Very low	Compared with the control, synbiotic iron supplementation may not importantly increase hemoglobin levels, although the evidence is very uncertain.
Presence of anemia	127 (1 RCT)	1/64 (1.6%)	4/63 (6.4%)	RR: 0.25 (0.03 to 2.14)	DR: −4.7 per 100 (−6 to +7)	⨁⨁◯◯^c^ Low	Compared to the iron control, iron-containing synbiotics may not produce an important effect on anemia.
Increase in hematocrit (%)	No studies						
Increase in ferritin (ng/ml)	208 (2 RCTs)	Average range: +0.4 to +7.76	Average range: −24.2 to +14.42	–	MD: + 2.03 (−2.79 to +6.84)	⨁⨁◯◯^b^ Low	Compared to the control, synbiotic iron does not importantly increase ferritin levels.
Adverse events	No adverse events were reported in one of the studies (Helmyati – 2020). Another study reported withdrawals for mild adverse events (gastrointestinal and taste acceptance) in the first few weeks, with no serious adverse events (Lovell – 2018).						

*In the Helmyati – 2020 study, the comparator group received iron as part of the control, while in the Lovell – 2018 study, the comparator group did not include iron supplementation.

RCT: Randomized Clinical Trial; HR: Hazard Ratio; CI: Confidence Interval; DR: Risk Difference; MD: Mean Difference

A MID of ≥1.0 g/dL was established for the increase in hemoglobin, 5 cases per 100 absolute difference for anemia, ≥ 3% for the increase in hematocrit, and ≥20 ng/ml for the increase in ferritin.

Explanations about the certainty of the evidence:

a. One level reduced the certainty due to risk of bias, since more than 30% of the weight of the meta-analysis is composed of studies with risk of bias.

b. Two levels reduced the certainty due to inaccuracy, since the sample is less than 30% (n = 240) of the OIS (n = 800).

c. Two levels reduced the certainty due to inaccuracy, since the 95% CI crosses two MIDs.

#### Prebiotics versus Placebo.

Compared to placebo, prebiotics do not produce an important effect on hemoglobin (5 RCTs; MD: + 0.15 g/dL; 95% CI: −0.37 to +0.67, high certainty), probably do not produce an important effect on the increase in hematocrit (4 RCTs; MD: −0.04%; 95% CI: −0.44 to +0.36, moderate certainty) and do not produce an important effect on the increase in ferritin (3 RCTs; MD: + 2.24 ng/ml; 95% CI: + 0.77 to +3.70, high certainty). In general, no serious adverse events were reported, although mainly mild gastrointestinal events were described (Table 1).

#### Prebiotics plus iron versus Standard control plus iron.

Compared to the iron control, prebiotics with iron probably do not produce an important effect on the increase in hemoglobin (4 RCTs; MD: −0.15 g/dL; 95% CI: −1.39 to +1.10, moderate certainty), may not produce an important effect on anemia (2 RCTs; RD: + 2 per 100; 95% CI: −9 to +21, low certainty) and do not produce an important effect on the increase in ferritin (2 RCTs; MD: −3.72 ng/ml; 95% CI: −8.55 to +1.11, high certainty). Prebiotics could have a protective effect against the adverse effects of iron on the microbiota and intestinal tract (Table 2).

#### Probiotics versus Placebo.

Compared with placebo, probiotics may not have an important effect on hemoglobin levels (3 RCTs; MD: + 0.17 g/dL; 95% CI: (−1.42 to +1.75; very low certainty). In addition, they do not produce an important effect on the increase in hematocrit (1 RCT; MD: 0.00%; 95% CI: −0.05 to +0.05, high certainty) and do not produce an important effect on the increase in ferritin (2 RCTs; MD: + 0.16 ng/ml; 95% CI: −6.76 to +7.09, high certainty). On the other hand, they could have an important effect on reducing anemia (1 RCT; RD: −6 per 100; 95% CI: −15 to +6, low certainty). The studies reported no adverse events associated with the intervention, although two deaths due to bronchopneumonia were observed in one trial (Dewan – 2007), not attributed to treatment (Table 3).

#### Probiotics plus iron versus Standard control plus iron.

Compared to the iron control, probiotics plus iron may not produce an important effect on the increase in hemoglobin (2 RCTs; MD: + 0.20 g/dL; 95% CI: −0.07 to +0.47, low certainty), may not produce an important effect on the increase in hematocrit (2 RCTs; MD: + 0.52%; 95% CI: −0.17 to +1.20, low certainty) and may not produce an important effect on the increase in ferritin (1 RCT; MD: −11.8 ng/ml; 95% CI: −20.29 to −3.31, very low certainty), although the evidence is very uncertain. None of the included studies reported information on adverse events (Table 4).

#### Synbiotics versus Placebo.

Compared to placebo, synbiotics do not produce an important effect on the increase in hemoglobin (2 RCTs; MD: −0.10 g/dL; 95% CI: −0.32 to +0.12, high certainty), probably do not produce an important effect on anemia (2 RCTs; RD: −2 per 100; 95% CI: −7 to +3, moderate certainty), do not produce an important effect on the increase in hematocrit (2 RCTs; MD: −0.04%; 95% CI: −0.34 to +0.27, high certainty) and do not produce an important effect on the increase in ferritin (1 RCT; MD: −0.27 ng/ml; 95% CI: −2.10 to +1.56, high certainty). In addition, the study of Xuan – 2013 found no differences in the percentage of change of hemoglobin [29]. Gastrointestinal adverse events and subclinical intestinal inflammation were reported without detailing frequency (Table 5).

#### Synbiotics plus iron versus Standard control with or without iron.

Compared with the control, with or without iron, synbiotics with iron may not importantly increase hemoglobin (2 RCTs; MD: + 0.23 g/dL; 95% CI: −0.09 to +0.56; very low certainty). Results were similar regardless of whether the comparator included iron. In addition, they may not have an important effect on anemia (1 RCT; RD: −4.7 per 100; 95% CI: −6 to +7, low certainty) and may not have an important effect on the increase in ferritin (2 RCTs; MD: + 2.03 ng/ml; 95% CI: −2.79 to +6.84, low certainty). Mild gastrointestinal adverse events were reported (Table 6).

## Discussion

In this systematic review with meta-analysis, we included RCTs evaluating the effects of prebiotics, probiotics, and synbiotics on hemoglobin and anemia in pediatric patients. Available evidence shows that prebiotics, probiotics, and synbiotics, alone or combined with iron, produce no important differences in hemoglobin, hematocrit, or ferritin levels compared to placebo or standard control (certainty: high to very low). Likewise, in most comparisons, no important reduction in anemia was observed; the only exception was probiotics versus placebo, which showed a important reduction in the risk of anemia (certainty: low). Regarding safety, no serious adverse events attributable to the interventions were reported, and the observed side effects were mainly mild and gastrointestinal.

The evidence included in this systematic review covers a large and heterogeneous pediatric population, ranging from neonates and infants to preschoolers, schoolchildren, and adolescents. Some RCTs focused on specific populations, such as children with malnutrition or anemia. At the same time, the majority included participants with and without baseline anemia, in whom iron doses administered as treatment or supplementation vary [16,41]. This clinical heterogeneity reflects real practice scenarios but also limits the extrapolation of observed effects to specific subgroups (patients with and without anemia). Notably, the consistency of the point estimates across trials spanning different age ranges, together with the absence of statistical heterogeneity, suggests that age did not meaningfully modify the efficacy of the interventions. A relevant gap is the lack of studies in hospitalized pediatric populations and in premature infants, groups with high nutritional vulnerability and increased risk of anemia, in which the response to prebiotics and probiotics may differ substantially [42,43]. However, the included studies are mainly representative of low- and middle-income countries, where the burden of childhood anemia is greater, thereby increasing their relevance for public health and applicability in contexts with a high prevalence of iron deficiency [44,45].

Regarding interventions, the most frequently evaluated prebiotics were galacto-oligosaccharides, oligofructose, fructo-oligosaccharides, and inulin. As for the probiotics, strains of the genus Lactobacillus predominated, administered alone or in combination with fructo-oligosaccharides. Interventions were evaluated against both placebo and in the context of fortified foods, with or without iron supplementation. Although there was substantial variability in dose, duration, and the specific type of prebiotic or probiotic, most outcomes did not show substantial heterogeneity. However, this apparent consistency does not exclude biological differences between strains or compounds, as previous evidence suggests that Lactobacillus plantarum, Lactobacillus acidophilus, Bifidobacterium infantis, and Bifidobacterium lactis may confer greater benefits in humans [8,46].

The evaluated outcomes included hemoglobin, anemia, hematocrit, ferritin, iron absorption, and adverse events. No randomized clinical trials directly evaluating iron absorption were identified. Although previous studies suggest that probiotics could improve intestinal iron absorption, this improvement does not necessarily translate into clinically relevant increases in hemoglobin, which is a more clinically important outcome in the pediatric population [47,48]. Regarding adverse events, most studies didn´t provide quantitative frequency data, limiting the evaluation of their magnitude.

Overall, the certainty of the evidence ranged from high to very low, depending on the type of intervention and the outcome evaluated. The risk of bias also contributed to decreased certainty in several comparisons, particularly those evaluating probiotics and probiotics plus iron, where a substantial proportion of the weight of the meta-analysis came from studies with methodological limitations, mainly related to incomplete outcome data, selective reporting of results, and other biases (baseline characteristic imbalances). Inconsistency was an uncommon reason for diminished certainty, as most studies found the intervention’s effect to be unimportant. Publication bias could not be quantitatively evaluated due to the small number of studies per meta-analysis; however, there was no qualitative suspicion of bias. There were also no problems of indirect evidence, as the studies were grouped by intervention.

A previous systematic review, last updated in 2023**,** evaluated the effects of prebiotics and probiotics on hemoglobin levels and iron absorption in children and women of reproductive age. They included 29 studies, of which 15 focused on children, in which a significant increase of 0.21 g/dL in hemoglobin was observed (8 studies; 95% CI 0.10 to 0.31, p < 0.0001). In contrast, the improvement in iron absorption was 3.72% (4 studies; 95% CI: −1.33 to 8.77), but this result was not statistically significant. This review used the GRADE approach, finding very low certainty for its results [16].

Another systematic review, with a search date of 2014, evaluated the effect of consuming fortified dairy products compared with cow’s milk on growth and nutritional status in children aged 6–47 months. Of the 15 articles, 4 included probiotics, prebiotics, or synbiotics in their interventions. A meta-analysis of 9 studies (1 study with synbiotics) showed a significant reduction in the risk of anemia (OR 0.32; 95% CI 0.15 to 0.66), with a greater effect in interventions lasting more than 7 months (OR 0.17; 95% CI 0.09 to 0.33). This review didn´t assess the certainty of the evidence using GRADE [17].

Another systematic review, published in 2019, evaluated the effects of probiotics on iron status across pediatric and adult populations. Of the 15 included studies, 8 were meta-analyzed, finding a significant increase in iron absorption with the use of L. plantarum 299 (SMD: 0.55; 95% CI: 0.22 to 0.88). However, hemoglobin results were not analyzed for heterogeneity between species. This review also didn´t use GRADE to evaluate the certainty of evidence [47].

This review provides a clearer picture of the available evidence by evaluating separate comparisons of prebiotics, probiotics, and synbiotics, with and without iron, exclusively in the pediatric population, and by applying the GRADE approach with MIDs. This made it possible to show that most of the interventions evaluated do not have a clinically important effect, offering a more rigorous and useful interpretation for decision-making.

The main strengths of this review include the registration of the protocol in PROSPERO, compliance with the PRISMA guidelines, the bibliographic search in relevant databases, the performance of the selection, extraction, and risk of bias processes independently, and the use of the GRADE approach to evaluate the certainty of the evidence using MIDs. In addition, we group the interventions to obtain more concrete and informative questions. On the other hand, gray literature was not searched, which may have led to the omission of relevant literature, although there was no qualitative suspicion of publication bias. In addition, prioritizing hemoglobin and anemia as the primary outcome may have led to the omission of studies that evaluated only secondary outcomes. On the other hand, the effect of the intervention was not investigated by dose, presentation, or duration; however, there was no heterogeneity in the vast majority of cases, suggesting that the interventions evaluated did not confer benefit regardless of these characteristics.

### Conclusion

In pediatric population, prebiotics, probiotics, and synbiotics, with or without iron, show no important benefit on hemoglobin, hematocrit, ferritin, or anemia, with high to very low certainty across comparisons and outcomes. On the other hand, probiotics versus a placebo could reduce the risk of anemia, but with low certainty. No serious adverse events attributable to the intervention were identified; the majority were mild gastrointestinal events. Higher-quality and sample-size clinical trials in high-risk populations are required to clarify potential specific benefits.

## Supporting information

S1 Checklist PRISMA 2020 checklist. (DOCX)

S2 Table Search strategy. (DOCX)

S3 Fig Flowchart for selecting the studies. (DOCX)

S4 Table Excluded studies reviewed in full text. (DOCX)

S5 Table Characteristics of the included studies (n = 19). (DOCX)

S6 Table Detailed characteristics of the intervention and comparator. (DOCX)

S7 Fig Risk of bias of the included studies. (DOCX)

S8 Table Adverse events. (DOCX)

S9 Fig Forest plots. (DOCX)
