# Supplementary material for: Efficacy and safety of prebiotics, probiotics, and synbiotics on hemoglobin and anemia in the pediatric population: A systematic review and meta-analysis
Source: PLoS One. 2026 Jul 29;21(7):e0354681. doi: 10.1371/journal.pone.0354681 (PMC13419176; doi:10.1371/journal.pone.0354681)
Supplement: S2 Table — (DOCX) [file pone.0354681.s002.docx]

Supplementary Table 2. Search strategy.

Search date: August 5, 2025

| Database | Search strategy | Results |
| --- | --- | --- |
| PubMed / MEDLINE | #1 Children  "Infant"[Mesh] OR "Child"[Mesh] OR "Adolescent"[Mesh] OR "Minors"[Mesh] OR "Pediatrics"[Mesh] OR Infant*[tw] OR child*[tw] OR adolescent *[tw] OR teen*[tw] OR youth*[tw] OR juvenile*[tw] OR "minor*"[tw] OR "Paediatr*"[tw] OR "Pediatr*"[tw] OR kid[tw] OR kids[tw]  #2 Prebiotics  "Prebiotics"[Mesh] OR prebiotic*[tw]  #3 Probiotics  "Probiotics"[Mesh] OR probiotic*[tw]  #4 Synbiotics  "Synbiotics"[Mesh] OR synbiotic*[tw]  #5 Anemia  "Anemia"[Mesh] OR "Hemoglobins"[Mesh] OR "Hematocrit"[Mesh] OR "Erythrocytes"[Mesh] OR anemia*[tw] OR anemia *[tw] OR hemoglobin*[tw] OR haemoglobin*[tw] OR ferrohaemoglobin *[tw] OR ferrohemoglobin *[tw] OR hematocrit*[tw] OR haematocrit *[tw] OR hemocrit [tw] OR Erythrocyte*[tw] OR “Red-Cell *”[tw] OR “blood cell*”[tw]  #6 Randomized controlled trial  ("randomized controlled trial"[pt] OR "controlled clinical trial"[pt] OR randomized[tiab] OR placebo [tiab] OR "clinical trials as topic"[mesh:noexp] OR randomly[tiab] OR trial[tiab]) NOT (animals [mh] NOT humans [mh])  #1 AND (#2 OR #3 OR #4) AND #5 AND #6 | 83 |
| CENTRAL  (via OVID) | "#1 Children  exp Infant/ OR exp Child/ OR exp Adolescent/ OR exp Minors/ OR exp Pediatrics/ OR Infant*.tw. OR Child*.tw. OR Adolescen*.tw. OR Teen*.tw. OR Youth*.tw. OR juvenil*.tw. OR Minor*.tw. OR Paediatr*.tw. OR Pediatr*.tw. OR kid.tw. OR kids.tw.    "#2 Prebiotics  exp Prebiotics/ OR prebiotic*.tw.    "#3 Probiotics  exp Probiotics/ OR probiotic*.tw.  "#4 Synbiotics  exp Synbiotics/ OR synbiotic*.tw.  "#5 Anemia"  exp Anemia/ OR exp Hemoglobins/ OR exp Hematocrit/ OR exp Erythrocytes/ OR anemia*.tw. OR anaemia*.tw. OR hemoglobin*.tw. OR haemoglobin*.tw. OR ferrohaemoglobin*.tw. OR ferrohemoglobin*.tw. OR hematocrit*.tw. OR haematocrit*.tw. OR hemocrit.tw. OR Erythrocyte*.tw. OR Red-Cell*.tw. OR "blood cell*".tw.  #1 AND (#2 OR #3 OR #4) AND #5 AND #6 | 141 |
| Embase | #1 Children  ‘Infant’/exp OR ‘Child’/exp OR ‘Adolescent’/exp OR ‘minor (person)’/exp OR ‘Pediatrics’/exp OR (Infant* OR Child* OR Adolescent * OR Teen* OR Youth* OR juvenile* OR ‘Minor*’ OR ‘Paediatr*’ OR ‘Pediatr*’ OR kid OR kids):ti,ab,kw    #2 Prebiotics  ‘prebiotic agent’/exp OR prebiotic*:ti,ab,kw    #3 Probiotics  ‘probiotic agent’/exp OR probiotic*:ti,ab,kw    #4 Synbiotics  ‘synbiotic agent’/exp OR synbiotic*:ti,ab,kw    #5 Anemia  ‘Anemia’/exp OR ‘Hemoglobin’/exp OR ‘Hematocrit’/exp OR ‘Erythrocyte’/exp OR (anemia* OR anaemia* OR hemoglobin* OR haemoglobin* OR ferrohaemoglobin * OR ferrohemoglobin * OR hematocrit* OR haematocrit * OR hemocrit OR Erythrocyte* OR ((Red OR blood) NEAR/2 Cell*)):ti,ab,kw    #6 Randomized controlled trial  ('randomized controlled trial'/de OR 'controlled clinical trial'/de OR random*:ti,ab,tt OR 'randomization'/de OR 'intermethod comparison'/de OR placebo:ti,ab,tt OR compare:ti,tt OR compared:ti,tt OR comparison:ti,tt OR ((evaluated:ab OR evaluate:ab OR evaluating:ab OR assessed:ab OR assess:ab) AND (compare:ab OR compared:ab OR comparing:ab OR comparison:ab)) OR ((open NEXT/1 label):ti,ab,tt) OR ((double OR single OR doubly OR singly) NEXT/1 (blind OR blinded OR blindly)):ti,ab,tt) OR 'double blind procedure'/de OR ((parallel NEXT/1 group*):ti,ab,tt) OR crossover:ti,ab,tt OR 'cross over':ti,ab,tt OR ((assign* OR match OR matched OR allocation) NEAR/6 (alternate OR groups OR intervention OR interventions OR patient OR subject OR subjects OR participants OR participants)):ti,ab,tt) OR assigned:ti,ab,tt OR allocated:ti,ab,tt OR ((controlled NEAR/8 (study OR design OR trial)):ti,ab,tt) OR volunteer:ti,ab,tt OR volunteers:ti,ab,tt OR 'human experiment'/de OR trial:ti,tt) NOT (((random* NEXT/1 sampl* NEAR/8 ('cross section*' OR questionnaire* OR survey OR surveys OR database OR databases)):ti,ab,tt) NOT ('comparative study'/de OR 'controlled study'/de OR 'randomised controlled':ti,ab,tt OR 'randomized controlled':ti,ab,tt OR 'randomly assigned':ti,ab,tt) OR ('cross‐sectional study' NOT ('randomized controlled trial'/de OR 'controlled clinical study'/de OR 'controlled study'/de OR 'randomized controlled':ti,ab,tt OR 'randomized controlled':ti,ab,tt OR 'control group':ti,ab,tt OR 'control groups':ti,ab,tt)) OR ('case control*':ti,ab,tt AND random*:ti,ab,tt NOT ('randomized controlled':ti,ab,tt OR 'randomized controlled':ti,ab,tt)) OR ('systematic review':ti,tt NOT (trial:ti,tt OR study:ti,tt)) OR (nonrandom*:ti, ab, tt not random *:ti,ab,tt NOT random*:ti,ab,tt OR 'random field*':ti,ab,tt OR (('random cluster' NEAR/4 sampl*):ti,ab,tt) OR (review:ab AND review:it)) NOT trial:ti,tt OR ('we searched':ab AND (review:ti,tt OR review:it)) OR 'update review':ab OR ((databases NEAR/5 searched):ab) OR ((rat:ti,tt OR rats:ti,tt OR mouse:ti,tt OR mice:ti,tt OR swine:ti,tt OR porcine:ti,tt OR murine:ti,tt OR sheep:ti,tt OR lambs:ti,tt OR pigs:ti,tt OR piglets:ti,tt OR rabbit:ti,tt OR rabbits:ti,tt OR cat:ti,tt OR cats:ti,tt OR dog:ti,tt OR dogs:ti,tt OR cattle:ti,tt OR cattle:ti,tt OR cattle:ti,tt OR monkey:ti,tt OR monkeys:ti,tt OR trout:ti,tt OR marmoset*:ti,tt) AND 'animal experiment'/de) OR ('animal experiment'/de not 'human experiment'/de))    #1 AND (#2 OR #3 OR #4) AND #5 AND #6 | 254 |
| Google Scholar | Prebiotic probiotic synbiotic children pediatric anemia hemoglobin randomized trial | 100 first results |
| Clinicaltrials.gov | #1 Children  Infant* OR Child* OR Adolescent* OR Teen* OR Youth* OR juvenile* OR Minor* OR Paediatr* OR Pediatr* OR kid OR kids    #2 Prebiotics, Probiotics, Synbiotics  prebiotic* OR probiotic* OR synbiotic*    #3 Anemia  anemia* or anaemia* or haemoglobin* or haemoglobin* or ferrohaemoglobin* or ferrohemoglobin* or hematocrit* or haematocrit* or Erythrocyte* or Red-Cell* or blood cell*  #1 AND #2  AND #3 | 47 |
