# Supplementary material for: Efficacy and safety of prebiotics, probiotics, and synbiotics on hemoglobin and anemia in the pediatric population: A systematic review and meta-analysis
Source: PLoS One. 2026 Jul 29;21(7):e0354681. doi: 10.1371/journal.pone.0354681 (PMC13419176; doi:10.1371/journal.pone.0354681)
Supplement: S3 Fig — (DOCX) [file pone.0354681.s003.docx]

# Supplementary Figure 3. Flowchart for selecting the studies.

Records identified from:

References of included articles (n =819)

Systematic Review References (n = 114)

**Identification of studies via databases and registers**

**Identification**

**Included**

Records screened by title-abstract

(n =492)

Records excluded

(n = 427)

Reports sought for retrieval

(n =65)

Reports not retrieved

(n = 0)

Reports assessed for eligibility

(n = 65)

Reports excluded (n= 46):

Wrong publication type (n= 14)

Ongoing RCTs (n= 10)

Wrong outcome (n= 17)

Wrong intervention (n = 2)

Wrong population (n= 2)

Duplicate record (n= 1)

Reports assessed for eligibility

(n= 6)

Studies included in review

(n = 19)

Reports of included studies

(n = 19)

Reports sought for retrieval

(n= 6)

Reports not retrieved

(n= 0)

Reports excluded (n=6):

Wrong population (n= 4)

Wrong outcome (n=1)

Duplicate record (n=1)

Records screened by title-abstract

(n= 1113)

Records excluded

(n= 1107)

**Screening**

**Identification of studies via other methods**

Records identified from all databases (n =625):

Embase (n = 254)

Ovid/CENTRAL (n = 141)

PubMed (n = 83)

Clinicaltrials.gov (n = 47)

Google Scholar (n = 100)

Records removed *before screening*:

Duplicate records removed manually using Rayyan Software (n = 133)
