# Supplementary material for: Efficacy and safety of prebiotics, probiotics, and synbiotics on hemoglobin and anemia in the pediatric population: A systematic review and meta-analysis
Source: PLoS One. 2026 Jul 29;21(7):e0354681. doi: 10.1371/journal.pone.0354681 (PMC13419176; doi:10.1371/journal.pone.0354681)
Supplement: S4 Table — (DOCX) [file pone.0354681.s004.docx]

Supplementary Table 4. Excluded studies were reviewed in full text.

| Author (year) | Title | Exclusion Reasons |
| --- | --- | --- |
| Jan  (2011) | Is the effect of probiotics on allergic rhinitis confined to Dermatophagoides farinae, Dermatophagoides pteronyssinus, or dust-sensitive children? A randomized, prospective, double-blind controlled trial | Outcomes Error |
| Khadilkar  (2023) | Proof of Concept Study to evaluate the effect of Iron and Prebiotic Supplementation in Children | Different publication type |
| Torkaman  (2017) | The effect of probiotics on reducing hospitalization duration in children with hyperbilirubinemia | Outcomes Error |
| Paganini  (2019) | Maternal human milk oligosaccharide profile modulates the impact of an iron and galacto-oligosaccharide intervention in Kenyan infants. | Outcomes Error |
| Giorgetti  (2023) | The effects of 2'-fucosyllactose and lacto-N-neotetraose, galacto-oligosaccharides, and maternal human milk oligosaccharide profile on iron absorption in Kenyan infants. | Outcomes Error |
| Scheuchzer (2024) | Iron Absorption from an Iron-Fortified Follow-Up Formula with and without the Addition of a Synbiotic or a Human-Identical Milk Oligosaccharide: A Randomized Crossover Stable Isotope Study in Young Thai Children. | Outcomes Error |
| Simran Sethi  (2025) | A clinical study to evaluate the effect of a test product (Lactic acid-based Probiotic tablet) for the treatment of Iron Deficiency Anemia in participants with Menorrhagia and Premenstrual Syndrome | Outcomes Error |
| Gerald Rosen  (2012) | Probiotics in the Treatment of Iron Deficiency in Children With Restless Leg Syndrome | Outcomes Error |
| NR  (2025) | Effect of Multi-Strain Probiotic on Ferritin Levels in Children with Iron Deficiency | RCT in progress |
| Jeannine Baumgartner (2021) | Potential of Prebiotic Galacto-oligosaccharides in Improving Efficacy and Safety of Oral Iron Supplementation in HIV-infected Children | RCT in progress |
| Anel Schoonees Martani J. (2020) | Probiotics in children with Severe Acute Malnutrition | Outcomes Error |
| But Cuicui (2024) | Clinical study on the use of animal Bifidobacterium lactis subspecies ProSci-246 to assist weight loss in obese school-age children by regulating gut microbiota. | RCT in progress |
| Chen Ke (2024) | Effect of New National Standard Infant Formula on the Growth and Development of Newborns with Cesarean Section. | RCT in progress |
| Michael Zimmermann (2014) | Iron and Prebiotics Fortification in Kenyan Infants | Different publication type |
| Michael Zimmermann (2017) | The Effect of the Prebiotic Galacto-oligosaccharide on Iron Absorption With Micronutrient Powders in Kenyan Infants. | Different publication type |
| Ying-Chen Lu (2022) | A double-blind, randomized, parallel, placebo-controlled study to evaluate the efficacy and safety of a probiotic containing Lactobacillus paracasei (eN-Lac®) for the treatment of children with perennial allergic rhinitis (year-round nose irritation caused by allergy). | Different publication type |
| Siti Helmyati, Lily A. Lestari (2018) | Effect of Double Fortification (Iron and Zinc) in Synbiotic Milk to Under 5 Years Stunted Children Growth. | RCT in progress |
| Kosek MN (2015) | Safety of Lactobacillus Reuteri in healthy children aged 2 to 5 years in Peru. | Outcomes Error |
| Amber Giorgetti, Nicole Stoffel (2020) | Prebiotic GOS and lactoferrin for beneficial gut microbiota with iron supplements (PREFER) | RCT in progress |
| Lambidou (2021) | Impact of an Infant Formula Containing a Novel Fat Blend (Cow's Milk Fat, Fish and Vegetable Oil) and Prebiotics on Stool Fatty Acid Soaps and Erythrocyte Fatty Acid Profiles in Full-Term Healthy Newborns. | Outcomes Error |
| Rosen (2019) | Use of a Probiotic to Enhance Iron Absorption in a Randomized Trial of Pediatric Patients Presenting with Iron Deficiency. | Outcomes Error |
| Scalabrin (2009) | Growth and tolerance of healthy term infants receiving hydrolyzed infant formulas supplemented with Lactobacillus rhamnosus GG: randomized, double-blind, controlled trial. | Outcomes Error |
| Rianda (2024) | The Effect of Probiotics and Calcium Supplementation During Childhood on Hemoglobin Level and Anemia Prevalence: A 10-Year Follow-Up Study | Different publication type |
| Wang (2024) | Clinical Efficacy of Modified Yigongsan Combined with Multi-enzyme Tablets and Bifidobacterium Triple Live Powder in Treatment of Infantile Anorexia with Spleen-Stomach Qi Deficiency Syndrome. | Different intervention |
| Saleem J, (2023) | Effects of Prebiotics on Metagenomic Diversity and Abundance in Young Children With Acute Malnutrition: A Double-blinded Randomised Controlled Trial (PMAM) | Different publication type |
| NR (2021) | Improving Efficacy and Safety of Oral Iron Supplementation in HIV-infected Children by Providing Prebiotic Galacto-oligosaccharides As Adjunct Treatment: a Randomized Controlled Trial. | RCT in progress |
| Habib-Ur-Rehman (2020) | Effects of galacto-oligosaccharide prebiotics on the blood profile of severely acute malnourished children. | Outcomes Error |
| NR (2019) | Celiac Disease and Vitamin Status: Evaluation of the Effect of Supplementation With a Probiotic (VIVOMIXX®) on Vitamin B6, B12, 25'OH D, Folic Acid, and Omocystein Levels, Metabolic and Inflammatory Status, and Gut Microbiota Metabolomics in a Cohort of Celiac Patients. | RCT in progress |
| NR (2019) | The Effect of Human Milk Oligosaccharides (HMOs) (2'-Fucosyllactose (2'-FL) and Lacto-N-neotetraose (LNnT)) and Galacto-oligosaccharides (GOS) on Iron Absorption From a Maize-based Porridge in Kenyan Infants | Outcomes Error |
| Mikulic (2021) | Consumption of a Single Dose of Prebiotic Galacto-Oligosaccharides Does Not Enhance Iron Absorption from Micronutrient Powders in Kenyan Infants: A Stable Iron Isotope Study. | Outcomes Error |
| Amber Giorgetti. (2019) | Prebiotic GOS and Lactoferrin for Beneficial Gut Microbiota With Iron Supplements | Duplicate |
| Souza (2018) | Randomized, double-blind, placebo-controlled parallel clinical trial assessing the effect of fructooligosaccharides in infants with constipation | Outcomes Error |
| NR (2018) | The Effect of Apo- and Holo-Lactoferrin and Dosing Regimen on Iron Absorption From a Maize-based Porridge in Kenyan Infants | Different publication type |
| Kortman (2018) | Iron fortification may reduce the efficacy of oral antibiotics against enteropathogens, whereas galacto-oligosaccharides (GOS) mitigate the adverse effects of iron fortification on the gut microbiome in Kenyan infants. | Different publication type |
| NR (2018) | Effect of Double Fortification (Iron and Zinc) in Synbiotic Milk on the Growth of Children under 5 Years: Stunted Children Growth: the Efforts to Achieve Target 2 SDGs Utilizing Local Natural Resources | Outcomes Error |
| Zimmermann (2017) | Improving safety and efficacy of iron fortification in Africa by combining iron and prebiotics: A stable isotope absorption study and a randomized controlled trial in Kenyan infants | Different publication type |
| Swarthout (2017) | Effect of probiotic administration on diarrhea incidence, severity, and the fecal microbiome in neonatal foals | Different population |
| Paganini (2017) | Effects of a micronutrient powder containing low-dose iron and galactooligosaccharides on the gut microbiome and iron absorption: A stable Iron isotope study and a randomized controlled trial in Kenyan infants | Different publication type |
| Harvey (2016) | Mineral status of infants requiring dietary management of cow's milk allergy by using an amino acid-based formula | Different publication type |
| NR (2016) | Testing Iron Absorption From a New Micronutrient Powder Containing Galacto-oligosaccharides (GOS) for Fortification of Infant Foods in Sub-Saharan Africa | Different publication type |
| Yan Liang (2015) | Study on Dietary Nutrition Intervention Techniques for Childhood Obesity | RCT in progress |
| NR (2014) | In Home Iron Fortification in Kenyan Infants: Effect of Co-supplementation With Galactooligosaccharides (GOS) on the Gut Microbiota Composition and the Effectiveness of Iron Supplementation | RCT in progress |
| Levinus Dieleman (2014) | Open-Label Trial of a Prebiotic Preparation Containing Inulin and Oligofructose (Synergy-1) for the Treatment of Mild to Moderate Acute Ulcerative Colitis | Different population type |
| Jin (2013) | Consuming a follow-up formula containing docosahexaenoic acid, prebiotics, and beta-glucan reduced the incidence and duration of acute respiratory infections in 3- to 4-year-old children. | Different publication type |
| Gerald Rosen  (2012) | Probiotics in the Treatment of Iron Deficiency in Children With Restless Leg Syndrome: A Double-blind, Randomized Controlled Study | Different publication type |
| Sazawal (2010) | Micronutrient fortified milk improves iron status, anemia, and growth among children 1-4 years: A double masked, randomized, controlled trial. | Different intervention |
