# Supplementary material for: Efficacy and safety of prebiotics, probiotics, and synbiotics on hemoglobin and anemia in the pediatric population: A systematic review and meta-analysis
Source: PLoS One. 2026 Jul 29;21(7):e0354681. doi: 10.1371/journal.pone.0354681 (PMC13419176; doi:10.1371/journal.pone.0354681)
Supplement: S5 Table — (DOCX) [file pone.0354681.s005.docx]

# Supplemental Table 5: Characteristics of the included studies (n=19).

| Author - year | Country | Population studied | Hb Inclusion Criteria | Sample size | Baseline characteristics (Hb g/dL, age, years, and sex) | Intervention | Control |
| --- | --- | --- | --- | --- | --- | --- | --- |
| Batool - 2023 | Pakistan | 6–59 months, severe acute malnutrition | NR | 213 | Hb 9.1 ± 1.3  Age 2.7 ± 1.1  Male 52.9% | Prebiotic | Standard Control/ Placebo |
| Feruś - 2018 | Poland | 4–18 years, celiac disease | NR | 34 | Hb 13.2 ± 1.0  Age 10.7 ± 1.0  Male 40.0% | Prebiotic | Standard Control/ Placebo |
| Li - 2014 | China | 3–4 years, daycare attendants | With and without anemia (Hb < 11 g/dL) | 310 | Hb 10.6 ± 0.8  Age 3.5 ± 0.3  Males NR  Anemia 65.0% | Prebiotic | Standard Control/ Placebo |
| Maximino - 2024 | Brazil | 3–12 months, born full term | With and without anemia (Hb < 11 g/dL) | 56 | Hb NR  Age 0.5 ± 0.2  Males 58.9%  Anemia 46.4% | Prebiotic | Standard Control/ Placebo |
| Bridges - 2016 | Brazil | 1–4 years, apparently healthy, cow's milk consumers | NR | 256 | Hb 11.7 ± 0.9  Age NR  Males NR | Prebiotic | Standard Control/ Placebo |
| Paganini - 2017a | Kenya | 6–14 months | Hb > 7 g/dL | 64 | Hb 10.5 ± 0.9  Age 0.8 ± 0.5  Male 48.0% | Prebiotic + iron | Iron control |
| Paganini - 2017b | Kenya | 6.5–9.5 months | Hb > 7 g/dL | 155 | Hb 10.4 ± 1.0  Age 0.8 ± 0.2  Male 50% | Prebiotic + iron | Standard Control/ Placebo and Iron Control |
| Putri - 2024 | Indonesia | 10–18 years, women with anemia | Anemia (Hb < 12 g/dL) | 61 | Hb 10.3 ± 1.2  Age NR  Males 0.0%  Anemia 100% | Prebiotic + iron | Iron control |
| Mikulic - 2019 | Kenya | 6–11 months, without acute or chronic diseases | Hb ≥ 7 g/dL | 191 | Hb 10.8 ± 1.0  Age 0.69 ± 0.11  Male 45.5% | Prebiotic (two arms according to dose) + iron | Iron control |
| Augustine - 2013 | Indonesia | 1–6 years, apparently healthy, no breastfeeding, low socioeconomic status | With and without anemia | 494 | Hb 11.9 ± 1.2  Age 4.9 ± 1.2  Male 54.5% | Probiotic (two probiotic arms) | Standard Control/ Placebo |
| Dewan - 2007 | India | 1–5 years, moderate to severe protein-energy malnutrition | NR | 80 | Hb 8.7 ± 1.6  Age 2.1 ± 0.9  Males NR | Probiotic | Standard Control/ Placebo |
| Mohammad - 2006 | Egypt | Average age: 11 years, apparently healthy | NR | 24 | Hb 12.1 ± 0.3  Age 11.1 ± 1.2  Males NR | Probiotic | Standard Control/ Placebo |
| Silva - 2008 | Brazil | 2–5 years, public nurseries | With and without anemia (Hb < 11 g/dL) | 190 | Hb 12.1 ± 0.7  Age 3.4 ± NR  Males NR  Anemia 16.6% | Probiotic + iron | Iron control |
| Manoppo - 2019 | Indonesia | 5–12 years, iron deficiency anemia | Iron Deficiency Anemia (Ret-He < 27.8 pg) | 68 | Hb 11.2 ± 2.5  Age 8.1 ± 4.6  Males NR  Anemia 100% | Probiotic + iron | Iron control |
| Sazawal - 2010 | India | 1–3 years, without severe malnutrition | Anemia (Hb < 10 g/dL) | 624 | Hb 9.1 ± 1.5  Age 1.9 ± 0.6  Male NR  Anemia 70.0% | Symbiotic | Standard Control/ Placebo |
| Kuitunen - 2009 | Finland | 0–2 years, mothers and their infants at high risk of allergies | NR | 781 | Hb NR  Age 1.3 ± NR  Male NR | Symbiotic | Standard Control/ Placebo |
| Xuan - 2013 | Vietnam | 18–36 months, healthy, no breastfeeding | NR | 368 | Hb 11.5 ± 1.0  Age 2.5 ± 0.5  Male 63.5% | Symbiotic | Standard Control/ Placebo |
| Helmyati - 2020 | Indonesia | 8–12 years, iron deficiency | NR | 59 | Hb 12.5 ± 1.0  Age NR  Males 39.0% | Symbiotic + iron | Iron control |
| Lovell - 2018 | New Zealand and Australia | 1 year, apparently healthy | Hb ≥ 10 g/dL | 160 | Hb 11.7 ± 0.8  Age NR  Males 62.5% | Synbiotic + iron | Standard Control/ Placebo |
