# Supplementary material for: Efficacy and safety of prebiotics, probiotics, and synbiotics on hemoglobin and anemia in the pediatric population: A systematic review and meta-analysis
Source: PLoS One. 2026 Jul 29;21(7):e0354681. doi: 10.1371/journal.pone.0354681 (PMC13419176; doi:10.1371/journal.pone.0354681)
Supplement: S6 Table — (DOCX) [file pone.0354681.s006.docx]

**Supplemental Table 6. Detailed characteristics of the intervention and comparator.**

| Author - year | Characteristics of the intervention (type, dose, frequency) | Adherence to the intervention | Control characteristics (type, dose, frequency) | Adherence to control | Funding |
| --- | --- | --- | --- | --- | --- |
| Batool - 2023 | Galacto-oligosaccharides (GOS; Vivinal® GOS) added to a therapeutic ready meal. Dose/duration: 4 g GOS/day for 8 weeks. Scheme by weight: 2 sachets/day (5–6.9 kg), 3 sachets/day (7–9.9 kg), 4 sachets/day (≥10 kg). | 100% | Standard control: Therapeutic food ready with starch (without prebiotic/probiotic). Dose/duration: 4 g/day for 8 weeks. | 100% | Open Access Publication Fund of Charité—Universitätsmedizin Berlin and the German Research Foundation (DFG). |
| Feruś - 2018 | Oligofructose + inulin. Dose/duration: 10 g/day (oral) for 3 months. | NR | Placebo/control: Maltodextrin (DE 20; Hotrimex, Konin, Poland). Dose/duration: 7 g/day (oral) for 3 months. | NR | Unfunded |
| Li - 2014 | Follow-up formula with DHA, polydextrose (PDX), galacto-oligosaccharides (GOS), and yeast β-glucan. Dose/frequency/duration: 3 servings/day for 28 weeks; each 40 g serving. | NR | Standard control: Cow's milk-based drink without DHA, prebiotics, or β-glucan. Dose/frequency/duration: 3 servings/day for 28 weeks; each serving 40 g of powder mixed with 200 ml of water. | NR | Mead Johnson Nutrition. |
| Maximino - 2024 | Cow's milk formula with galacto-oligosaccharides (GOS) and fructo-oligosaccharides (FOS). Dose/frequency/duration: median of 437 mL/day (GOS 2.6 g and FOS 0.29 g per 100 g) for 24 weeks. | NR | Goat's milk formula. Dose/frequency/duration: median of 412 mL/day for 24 weeks. | NR | Ausnutria BV. |
| Bridges - 2016 | Cow's milk drink with polydextrose (1.2 g/serving), galacto-oligosaccharides (1.2 g/serving), and 8.7 mg of yeast β-glucan. Dose/frequency/duration: 3 servings/day for 28 weeks. | NR | Cow's milk drink without supplementation. Dose/frequency/duration: 3 servings/day for 28 weeks. | NR | Mead Johnson Nutrition. |
| Paganini - 2017a | Micronutrient powder with galacto-oligosaccharides (GOS; Vivinal® GOS 75 Powder) + iron (ferrous fumarate + NaFeEDTA). Composition/dose: 7.5 g GOS/day + NaFeEDTA 2.5 mg + ferrous fumarate (for a total of 5 mg Fe/day). Frequency/duration: 1 sachet/day for 5 weeks. | 98% | Control (micronutrient without GOS) with iron. Frequency/duration: 1 sachet/day for 5 weeks. Reported composition: maltodextrin 10.5 g; iron 2.5 mg as ferrous fumarate + 2.5 mg as NaFeEDTA; vitamin C 30 mg. | 98% | ETH Global, ETH Zurich, and DSM Nutritional Products funded the study. Friesland-Campina donated the galactooligosaccharides used in the study. |
| Paganini - 2017b | Micronutrient powder (MNP) with iron + galacto-oligosaccharides (GOS). Composition: iron 5 mg/day (2.5 mg as NaFeEDTA + 2.5 mg as ferrous fumarate) + 10.5 of 75% of GOS (Vivinal® GOS 75 Powder). | 94% | Two control groups: (1) MNP without iron or GOS: ascorbic acid 30 mg, vitamins and minerals, microbial phytase 190 FTU (Tolerase 20000G), and maltodextrin 10.5 g; (2) MNP with iron: composition identical to control (1) + iron 5 mg/day (2.5 mg NaFeEDTA + 2.5 mg ferrous fumarate). | Iron-free 94%; with iron 95% | ETH Global and the Sawiris Foundation for Social Development; ETH Zurich, Switzerland; DSM Nutritional Products, Kaiseraugst, Switzerland. Sight and Life (Kaiseraugst, Switzerland) donated the MNPs used in this study. FrieslandCampina (Wageningen, Netherlands). |
| Putri - 2024 | Mung bean drink with inulin + iron tablets. Dose/frequency/duration: Mungo (40 g/250 ml) with inulin 10 g/day + 1 tablet/day of elemental iron (60 mg) + 400 mcg of folic acid for 12 weeks. | NR | Palm sugar drink + iron tablets. Dose/frequency/duration: 1 bottle/day + 1 tablet/day of elemental iron (60 mg) for 12 weeks. | NR | Ministry of Research and Technology/National Agency for Research and Innovation of the Republic of Indonesia. |
| Mikulic - 2019 | Iron fumarate with galacto-oligosaccharides / fructo-oligosaccharides. Dose/frequency/duration: 2 servings/day for 3 weeks (each serving: 7.5 g or 3 g with 3.6 mg of iron). | 93% (7.5 g + iron); 90% (3 g + iron) | Iron fumarate. Dose/frequency/duration: 2 servings/day (3.6 mg) for 3 weeks. | 87% | Danone Research and ETH Zurich, Switzerland. |
| Augustine - 2013 | Regular milk with calcium supplemented with Lactobacillus reuteri DSM 17938 or Lactobacillus casei CRL 431. Dose/frequency/duration: 5 × 10⁸ CFU/day for 6 months. (Study with two probiotic arms) | 94% | Low-lactose milk administered twice daily (180 mL per intake), unaccompanied by meals, with low (~50 mg/day; LC group) or regular calcium content (~440 mg/day; RC group). (Study with two control arms) | 94% | Top Institute Food and Nutrition, Wageningen; FrieslandCampina; and Unilever Research and Development, Netherlands. The International Nutrition Foundation in the USA provided a doctoral fellowship for the first author. No funding was obtained from the manufacturers that provided the probiotic strains. |
| Dewan - 2007 | Protein concentrate with Lactobacillus bulgaricus and Streptococcus thermophilus. Dose/frequency/duration: 200 g/day in 2 servings of 100 g. 1 g of curd contained 10⁸ CFU of L. bulgaricus and S. thermophilus, administered from day 7 to day 15. | NR | Berseem Leaf Protein Concentrate  Dose/frequency/duration: 3 sachets per day with a supply of 6 g of protein/day, from day 7 to day 15. | NR | Indian National Science Academy (India National Science Academy). |
| Mohammad - 2006 | Yogurt with Lactobacillus acidophilus. Dose/frequency/duration: 2 cups of 5 x 10 ^9^ CFU/ day (75 g/day) for 42 days. | NR | Commercial Yogurt. Dose/frequency/duration: daily consumption for 42 days. | NR | NR |
| Silva - 2008 | Culture of Streptococcus thermophilus + Lactobacillus bulgaricus + culture of Lactobacillus acidophilus + iron amino acid chelate. Dose/frequency/duration: 80 mL/day, providing 10⁸ CFU/ml of L. acidophilus + 3 mg of iron per dose, from Monday to Friday for 101 days of class. | NR | Iron milk without probiotics. Dose/frequency/duration: 80 mL per dose (3 mg of iron), from Monday to Friday for 101 days of class. | NR | NR |
| Manoppo - 2019 | Iron with Lactobacillus reuteri DSM 17938 (3 × 10⁸ CFU/day). Duration: 14 days. | NR | Ferrous sulphate in tablets. Dose/frequency/duration: 2 tablets/day (120 mg) for 14 days. | NR | NR |
| Sazawal - 2010 | Milk fortified with Bifidobacterium lactis HN019 (1.9 × 10⁷ CFU/day) + prebiotic oligosaccharides (2.4 g/day). Duration: 12 months. | >85% | Standard control: Milk powder similar in composition, appearance, size, taste, and packaging, without probiotics or prebiotics. Dose/frequency/duration: 32 sachets of 32 g for 12 months. | >85% | Anchor Institute, part of Fonterra Brands, Singapore. |
| Kuitunen - 2009 | Probiotic mixture Lactobacillus rhamnosus GG (ATCC 53103) 5 × 10  CFU, L. rhamnosus LC705 (DSM 7061) 5 × 10  CFU, Bifidobacterium breve Bb99 (DSM 13692) 2 × 10  CFU, Propionibacterium freudenreichii ssp. shermanii JS (DSM 7076) 2 × 10  CFU, + galacto-oligosaccharides (GOS) 0.8 g/day. Scheme: daily from 4 weeks before delivery to 6 months of age. | NR | Placebo: Microcrystalline cellulose + sugar syrup, without oligosaccharides. Scheme: from 4 weeks before delivery and after birth to 6 months of age. | NR | Helsinki University Central Hospital Research Funds, the Foundation for Pediatric Research, Helsinki, Finland, and Valio Ltd, Helsinki, Finland. M.K. received a part-time salary from Helsinki University Central Hospital Research Funds. K.K. received a salary from the Clinical Research Institute of Helsinki University Central Hospital Ltd, funded by Valio Ltd. |
| Xuan - 2013 | Growth milk with Lactobacillus paracasei and Bifidobacterium longum (10⁸ CFU/day) + inulin and fructo-oligosaccharides (2.2 g/day). Dose/frequency/duration: administration 5 times a week for 5 months. | NR | Growth milk without supplementation with probiotics or prebiotics. Dose/frequency/duration: 360 mL/day, 5 times a week for 5 months. | NR | Nestlé research fund. |
| Helmyati - 2020 | Milk fermented with Lactobacillus plantarum (10⁸–10⁹ CFU/mL) + fructo-oligosaccharides (5 g per cup) + iron syrup. Dose/frequency/duration: 1 tablespoon/day with 10 mg of elemental iron, administered 2 times/week for 3 months. | 99.35% (L. plantarum); 89.33% (iron syrup) | Iron syrup. Dose/frequency/duration: 1 tablespoon with 10 mg of elemental iron, 2 times/week for 3 months. | 94.83% | Nestlé Foundation. |
| Lovell - 2018 | Cow's milk fortified with iron, galacto-oligosaccharides (GOS), and fructo-oligosaccharides (FOS). Dose/frequency/duration: GOS 1.8 g/day + FOS 0.2 g/day + iron (1.7 mg/100 mL) for 12 months. | 89% | Unfortified cow's milk. Dose/frequency/duration: 300 mL/day for 12 months. Composition: iron 0.02 mg/100 mL and cholecalciferol 0.06 µg/100 mL. | 89% | Danone Pty. Ltd. and Danone Nutricia Research. |
