# Supplementary figures and images for: Efficacy and safety of prebiotics, probiotics, and synbiotics on hemoglobin and anemia in the pediatric population: A systematic review and meta-analysis
Source: PLoS One. 2026 Jul 29;21(7):e0354681. doi: 10.1371/journal.pone.0354681 (PMC13419176; doi:10.1371/journal.pone.0354681)

# Supplemental Figure 7. Risk of bias of the included studies.


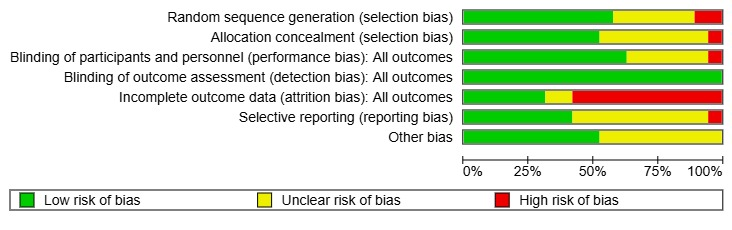

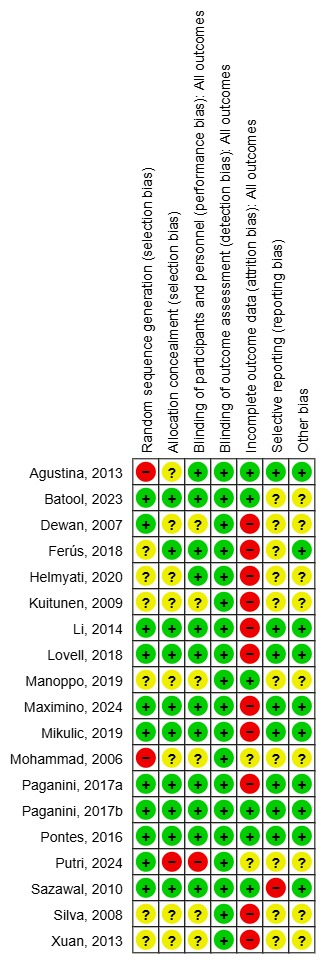

Supplement: S7 Fig — (DOCX) [file pone.0354681.s007.docx]
