# Supplementary material for: Efficacy and safety of prebiotics, probiotics, and synbiotics on hemoglobin and anemia in the pediatric population: A systematic review and meta-analysis
Source: PLoS One. 2026 Jul 29;21(7):e0354681. doi: 10.1371/journal.pone.0354681 (PMC13419176; doi:10.1371/journal.pone.0354681)
Supplement: S8 Table — (DOCX) [file pone.0354681.s008.docx]

# Supplemental Table 8. Adverse Event Table.

| Study - year | Adverse events reported |
| --- | --- |
| Batool – 2023 | No real or suspected adverse events were reported (0 events). |
| Ferus – 2018 | No serious adverse events were observed; frequency of symptoms with no significant differences between groups (no figures). |
| Li – 2014 | 1 case of allergic manifestation (food allergy). |
| Maximino – 2024 | Mild GI symptoms: crying >1 h/day 7% (80 episodes), regurgitation >2/day 13% (165 episodes), watery/hard stools 6% (71 episodes); no relevant differences between groups; lower risk of regurgitation in CMF (OR 0.10; 95% CI 0.02–0.68). |
| Pontes – 2016 | Oral candidiasis: 5 cases in LBCM versus 0 in control (p = 0.03); severe AEs: 10 in control versus 2 in LBCM. |
| Paganini – 2017a | NR. |
| Paganini – 2017b | DOGS mitigated the adverse effects of iron; effects of iron alone (microbiome alterations, intestinal damage, respiratory infections, inflammation, and diarrhea) are described, without quantification. |
| Putri – 2024 | NR; protective effect is mentioned (no figures). |
| Mikulic – 2019 | The scGOS/lcFOS mixture mitigated the adverse effects of iron on the microbiome (no figures). |
| Agustina – 2013 | No adverse events were reported; 0 events related to cow's milk protein. |
| Dewan – 2007 | 2 deaths from bronchopneumonia (1 per group), not attributable to the intervention. |
| Mohammad – 2006 | NR. |
| Silva – 2008 | NR. |
| Manoppo – 2019 | NR. |
| Sazawal – 2010 | No adverse events were reported during 1 year of follow-up (0 events). |
| Kuitunen - 2009 | Mild subclinical intestinal inflammation (fecal↑ calprotectin and α1-antitrypsin; no figures). |
| Xuan – 2013 | Adverse events were recorded, mainly gastrointestinal (no figures or detailed severity). A trend was observed, feeding GAU 1 milk reduced the incidence of all categories of adverse events, including respiratory and gastrointestinal events. |
| Helmyati – 2020 | NR. |
| Lovell – 2018 | Withdrawals for mild AEs (GI/taste acceptance) in the first few weeks; 0 serious adverse events. |
