# Supplementary material for: Efficacy and safety of prebiotics, probiotics, and synbiotics on hemoglobin and anemia in the pediatric population: A systematic review and meta-analysis
Source: PLoS One. 2026 Jul 29;21(7):e0354681. doi: 10.1371/journal.pone.0354681 (PMC13419176; doi:10.1371/journal.pone.0354681)

# Supplemental Figure 9. Forest plots.

Prebiotics versus Placebo.

a. Increase in hemoglobin (%).


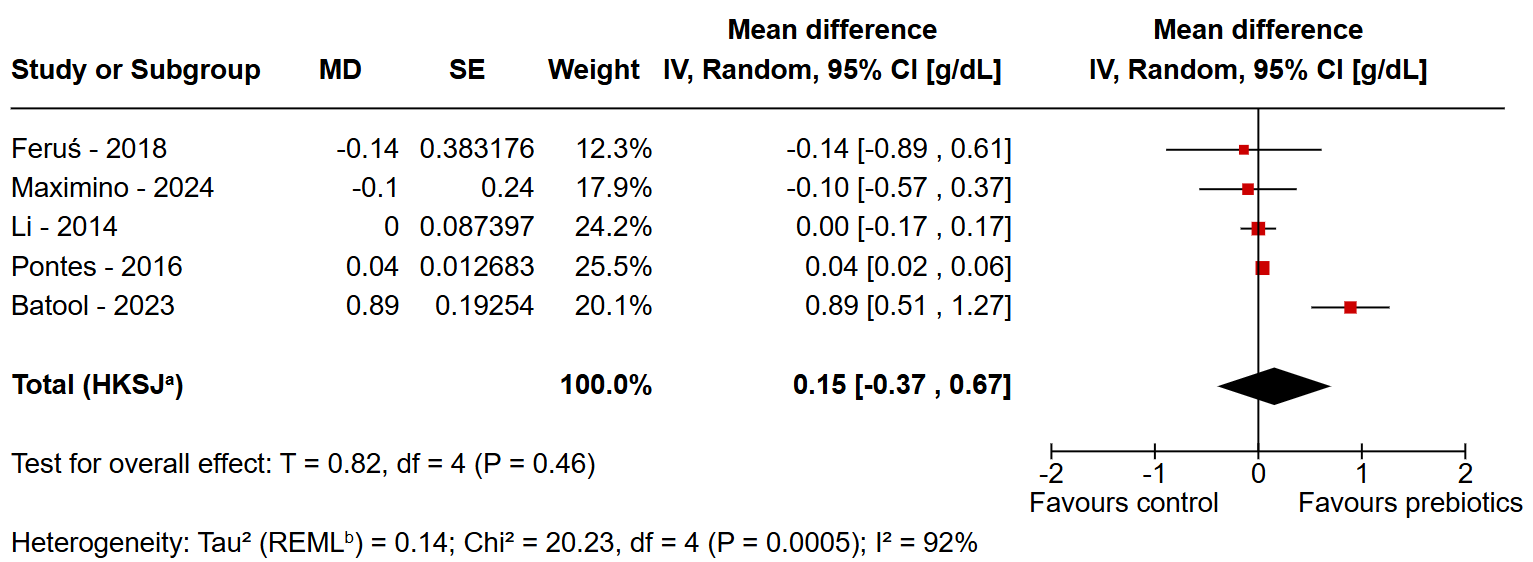


b. Increase in hematocrit (%).


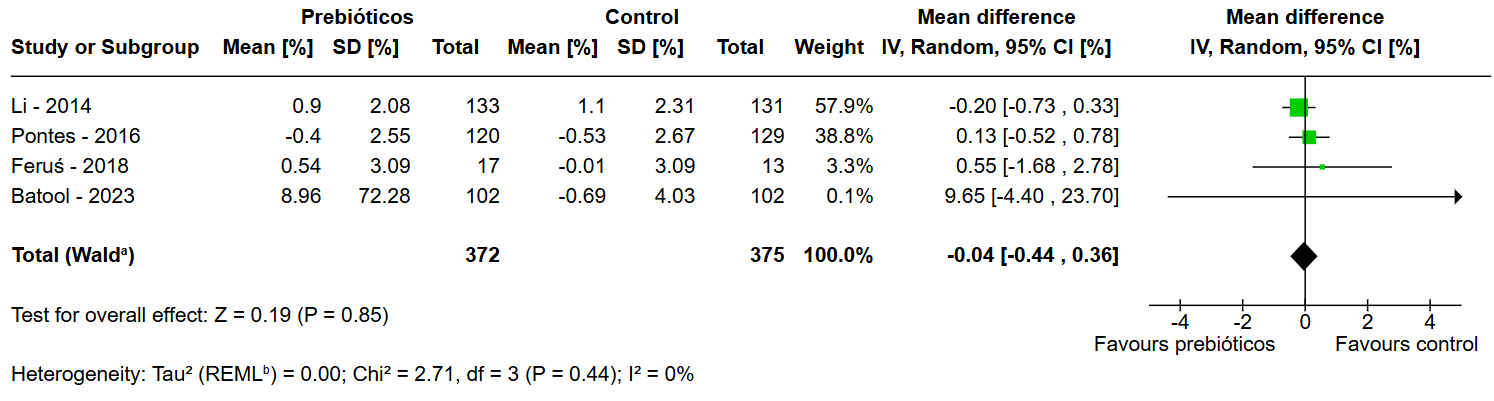


c. Increase in ferritin (ng/ml).


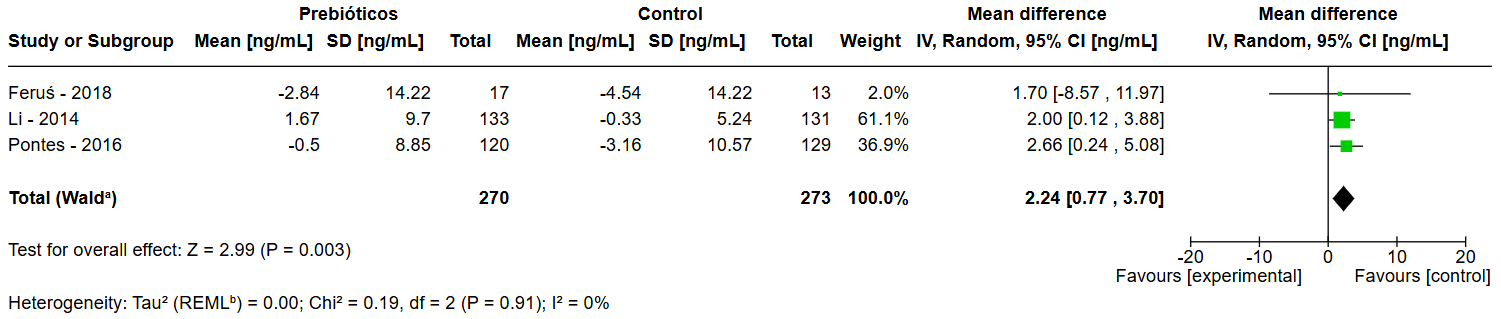


Prebiotics plus iron versus standard control plus iron.

a. Increase in hemoglobin (%).


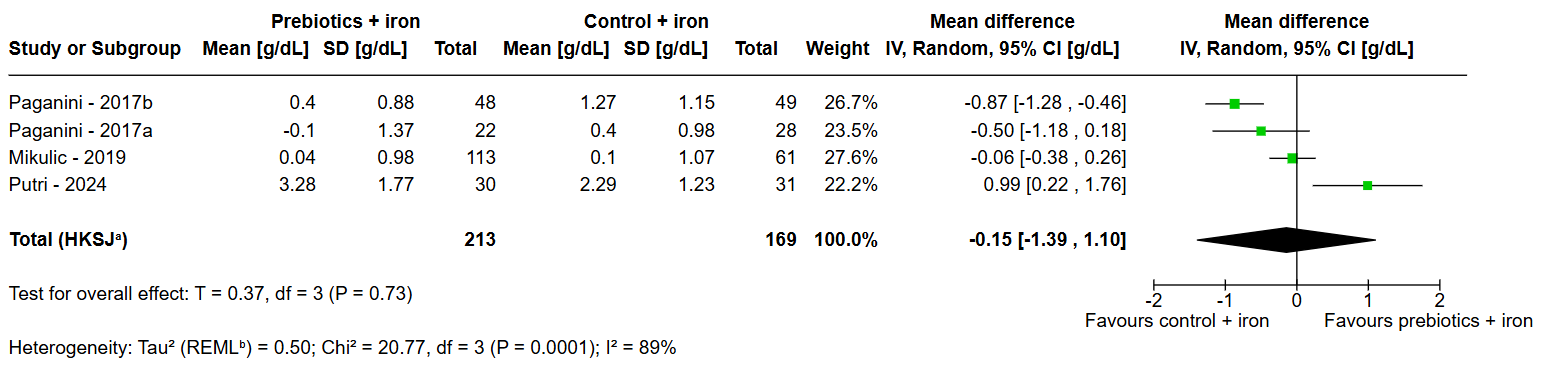


b. Presence of anemia.


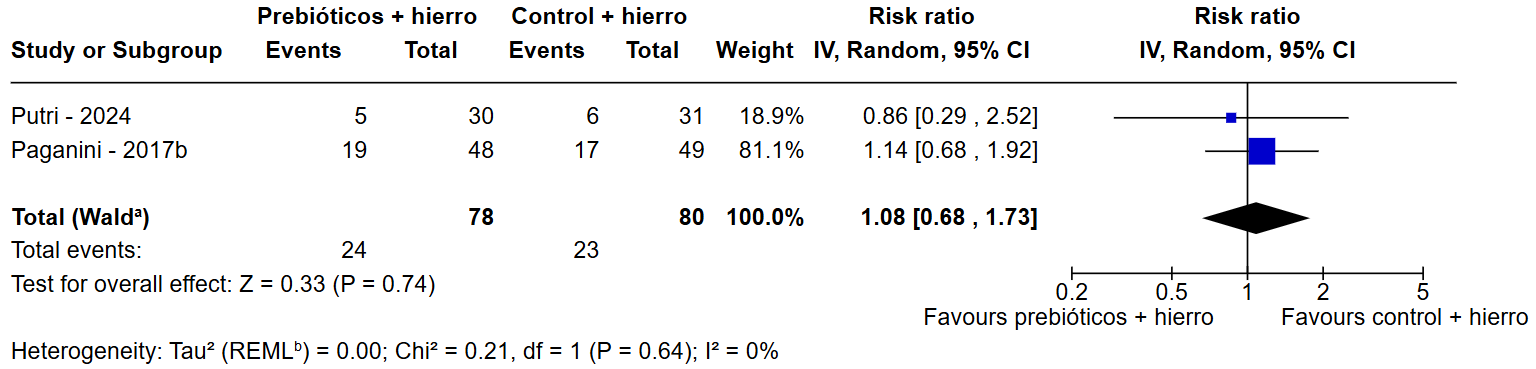


c. Increase in ferritin (ng/ml).


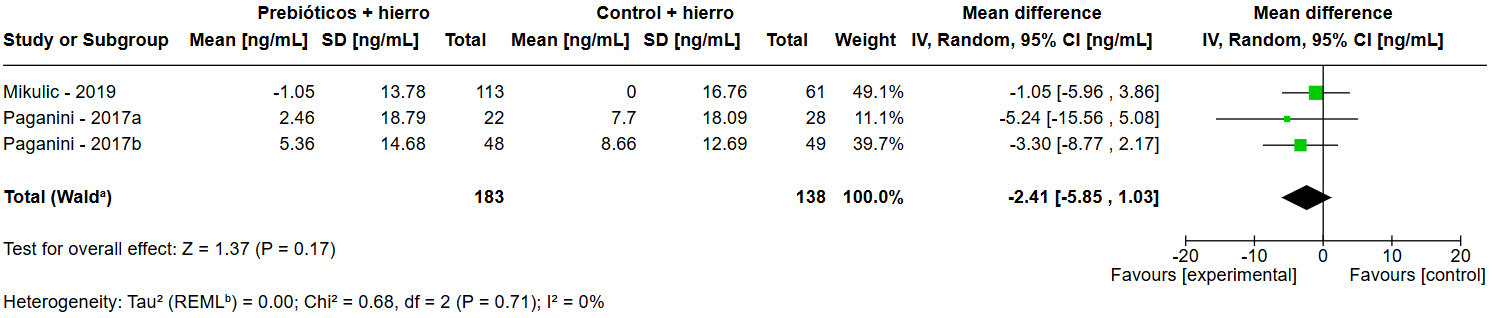


Probiotics versus Placebo

a. Increase in hemoglobin (%).


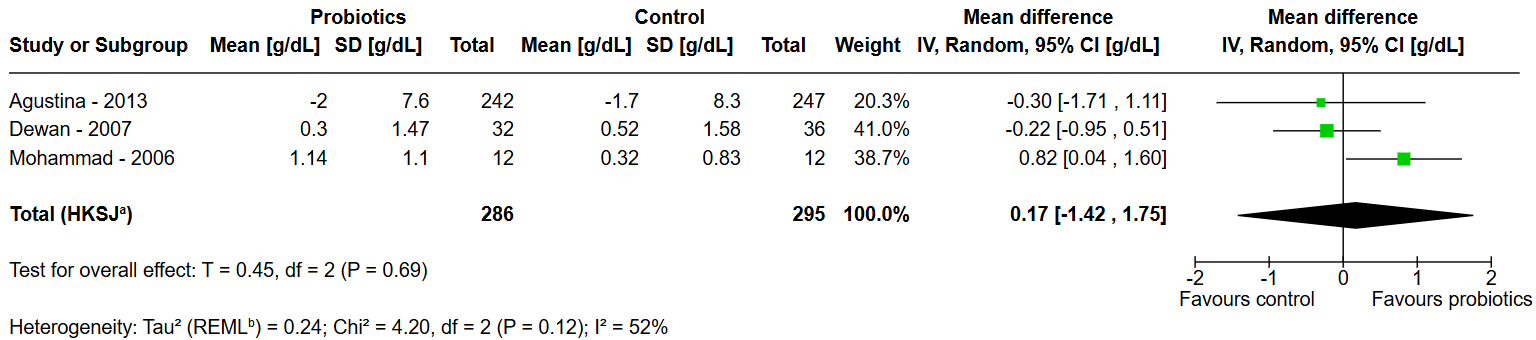


b. Increase in ferritin (ng/ml).


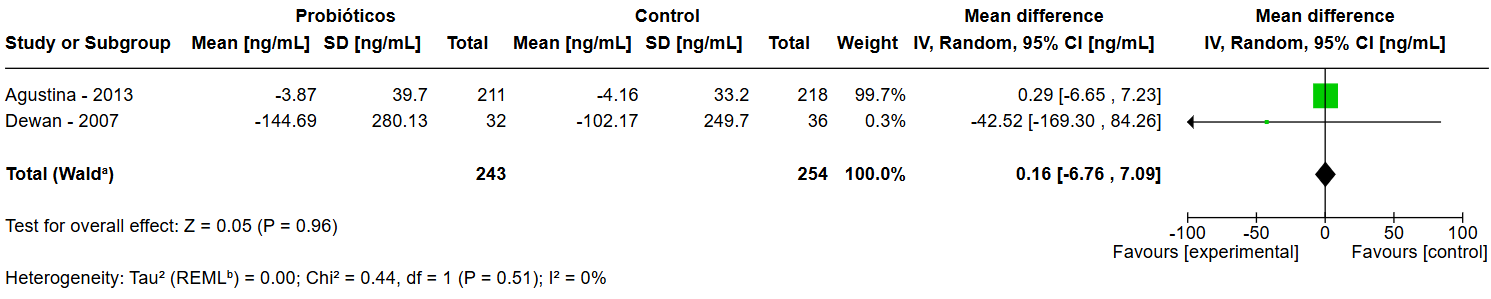


Probiotics plus iron versus standard control plus iron.

a. Increase in hemoglobin (%).


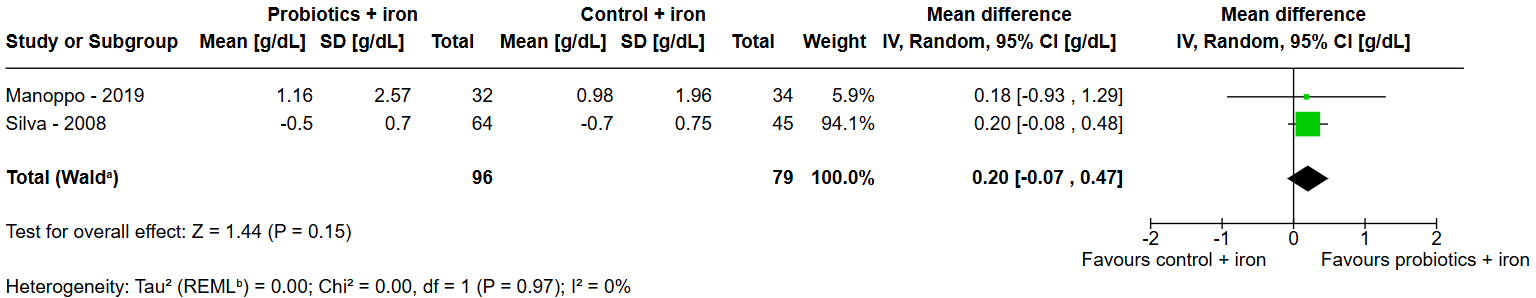


b. Increase in hematocrit (%).


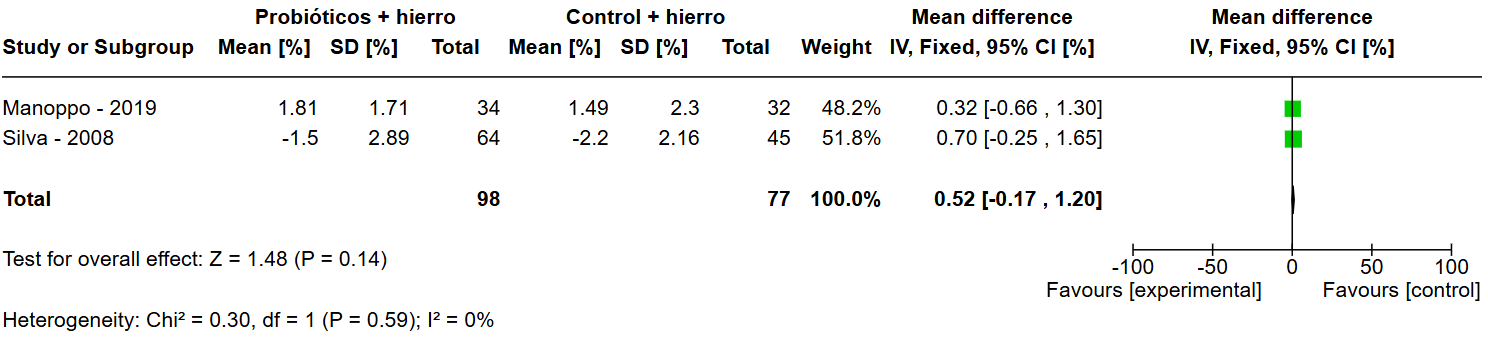


Symbiotics versus Placebo.

a. Increase in hemoglobin (%).


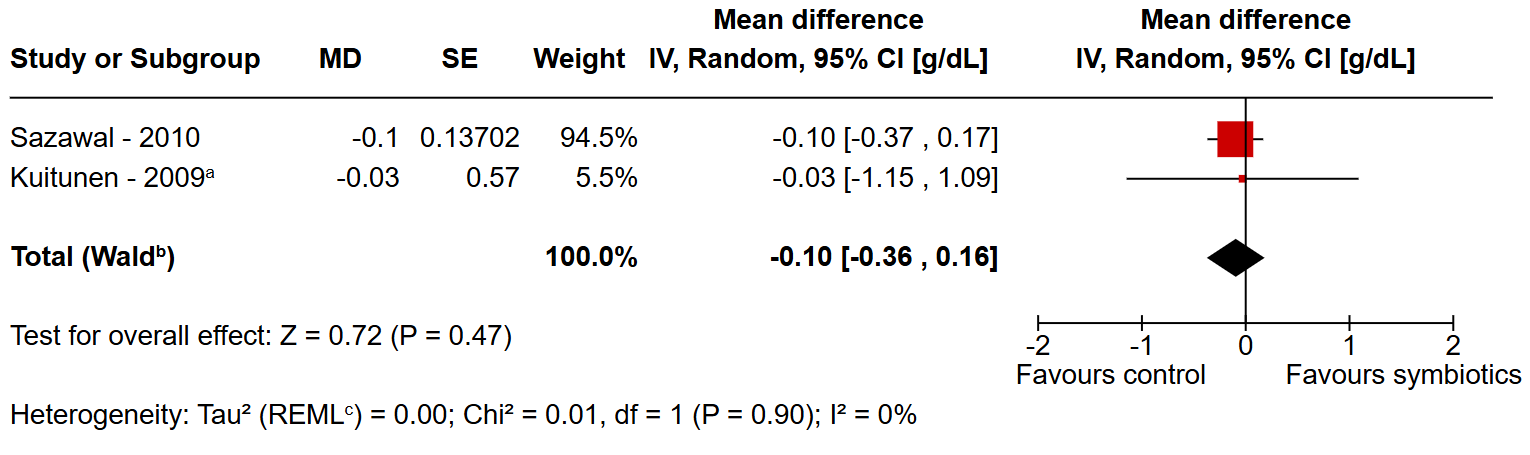


b. Presence of anemia.


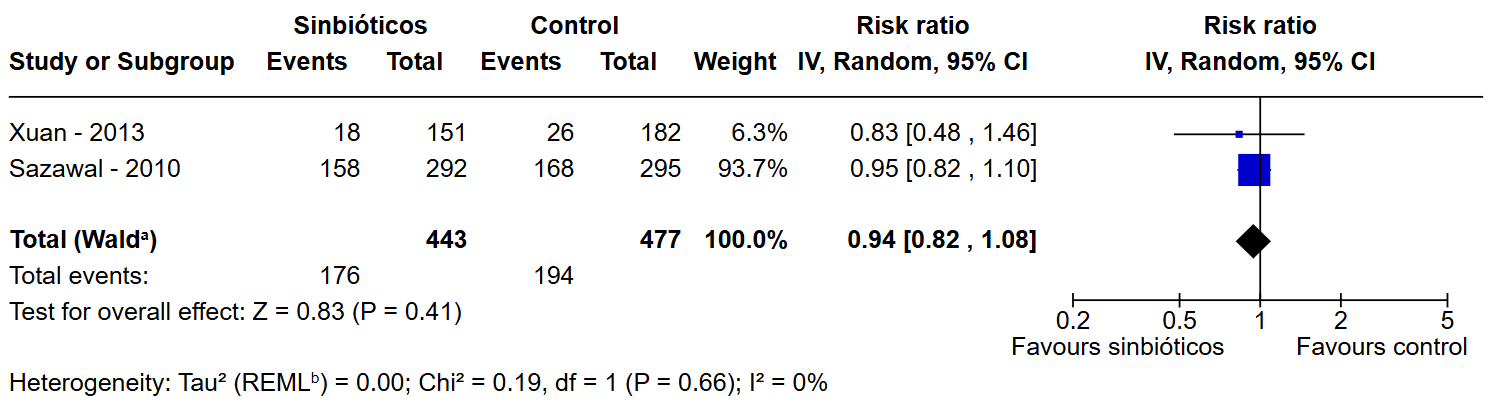


c. Increase in hematocrit (%).


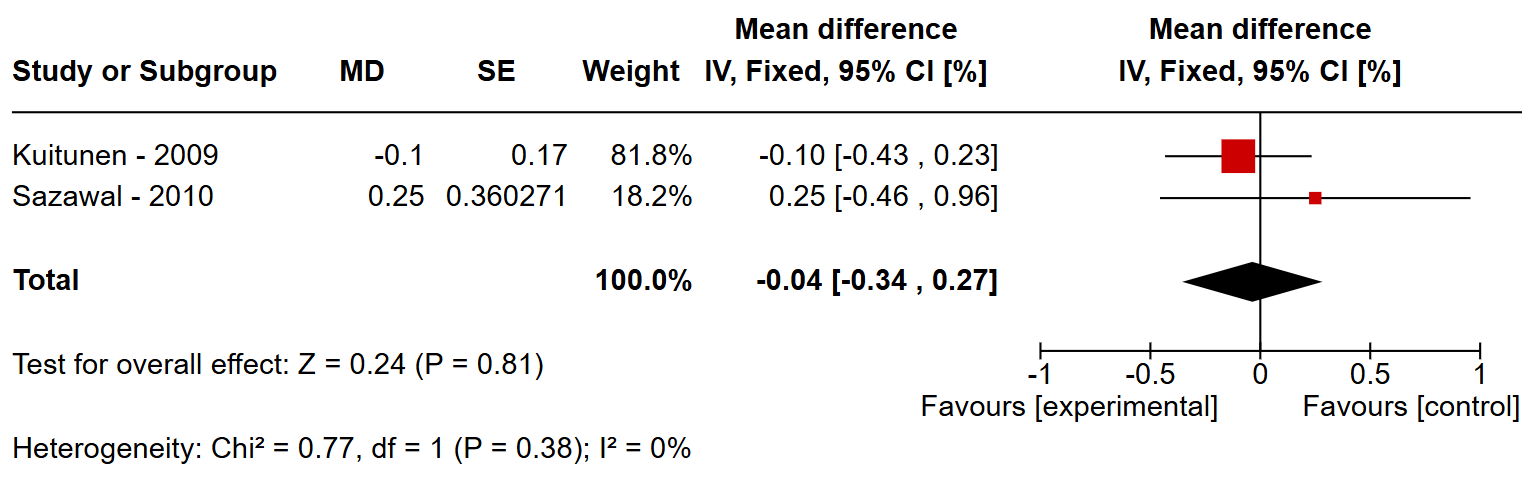


Symbiotics plus iron versus standard control with or without iron

a. Increase in hemoglobin (%).


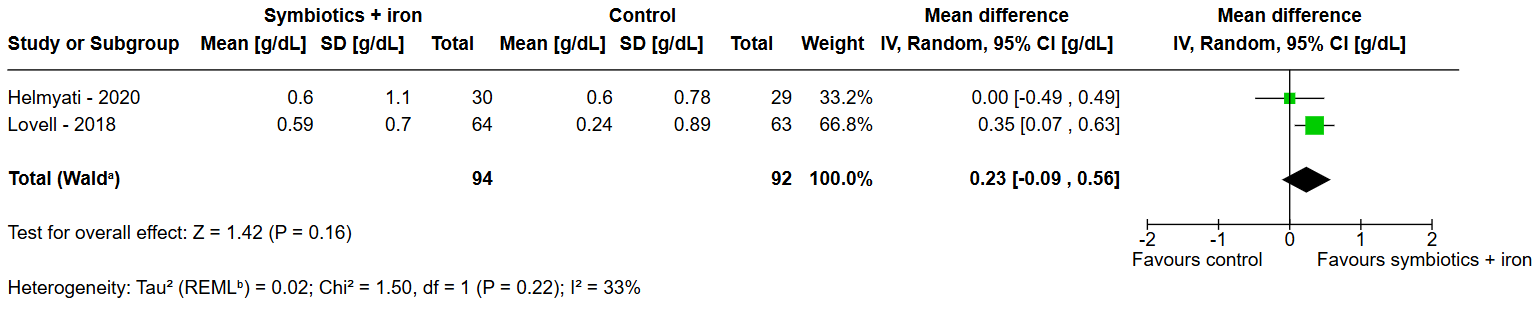


b. Increase in ferritin (ng/ml).
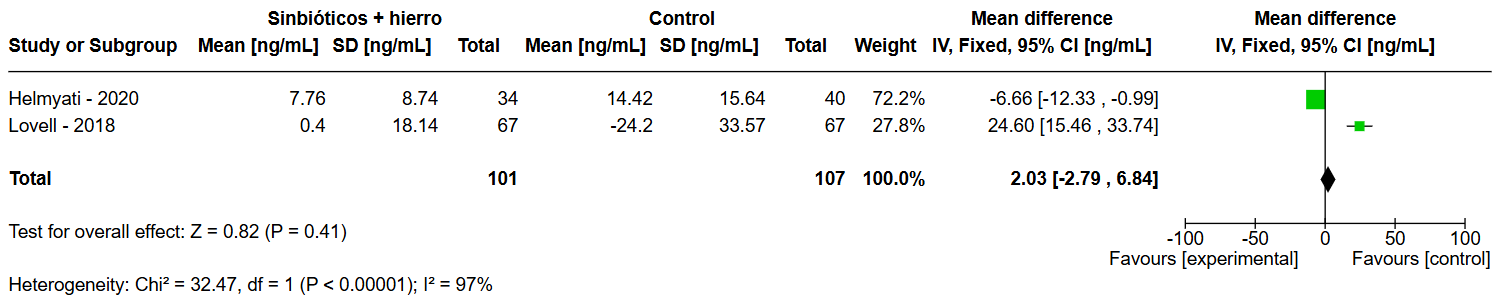

Supplement: S9 Fig — (DOCX) [file pone.0354681.s009.docx]
