# Supplementary material for: Efficacy and safety of prebiotics, probiotics, and synbiotics on hemoglobin and anemia in the pediatric population: A systematic review and meta-analysis
Source: PLoS One. 2026 Jul 29;21(7):e0354681. doi: 10.1371/journal.pone.0354681 (PMC13419176; doi:10.1371/journal.pone.0354681)
Supplement: Attachment — Submitted filename: Review_PLOS One manuscript.docx [file pone.0354681.s010.docx]

**Title: Efficacy and safety of prebiotics, probiotics, and symbiotics on hemoglobin and anemia in the pediatric population: A systematic review and meta-analysis**

The authors have followed established methodological standards, including PRISMA, GRADE, and PROSPERO registration, and have clearly communicated the findings.

Instead of focusing only on statistical significance, the authors defined Minimal Important Differences (MIDs) for each outcome in advance and used these to interpret the findings. This is a thoughtful and rigorous approach that strengthens the interpretation of the results.

**A few comments:**

1. The comparison ‘synbiotics plus iron vs. standard control with or without iron’ pools two fundamentally different comparators: one where the control arm receives iron and one where it does not. The clinical heterogeneity is not adequately addressed. The footnote acknowledges this but the justification for pooling is missing.

A sensitivity analysis excluding the study with a non-iron comparator could be considered and the reasons why the pooled estimate remained informative could be discussed.

2. Several outcomes are based on only one RCT, yet conclusions have been drawn from them:

• Anemia with probiotics vs. placebo- 1 RCT (in table 3 and in the discussion & conclusion)

• Hematocrit with probiotics vs. placebo- 1 RCT (in table 3 and results section)

• Anemia with synbiotics + iron-1 RCT (in table 6 and results section)

The single-study findings are presented in the same format as meta-analysis findings and carried forward into the abstract & conclusion as a noteworthy result. However, they should be clearly distinguished as single-study findings from pooled estimates or preliminary, single-trial evidence.

3. The included studies heterogenous pediatric age range from 0.5 years to ~11 years. No subgroup or sensitivity analysis was performed by age group. Given that iron physiology, gut microbiome composition, and intervention responsiveness differ substantially across early infancy, childhood, and adolescence, this is an important limitation. This has been mentioned in the discussion, but not adequately addressed why subgroup analysis was not performed.

4. Table 3 is titled ‘Summary of results of the effects of prebiotics versus placebo’, but it clearly reports probiotics data. This must be corrected.
