# Supplementary material for: Efficacy and safety of prebiotics, probiotics, and synbiotics on hemoglobin and anemia in the pediatric population: A systematic review and meta-analysis
Source: PLoS One. 2026 Jul 29;21(7):e0354681. doi: 10.1371/journal.pone.0354681 (PMC13419176; doi:10.1371/journal.pone.0354681)
Supplement: Attachment — Submitted filename: Response to reviewers - COMPLETED.docx [file pone.0354681.s011.docx]

**RESPONSE TO REVIEWERS**

**Manuscript title:** Efficacy and safety of prebiotics, probiotics, and synbiotics on hemoglobin and anemia in the pediatric population: A systematic review and meta-analysis

**Manuscript ID:** PONE-D-26-15986

We sincerely thank both reviewers for their careful, rigorous, and constructive evaluation of our manuscript. Their comments have helped us improve the clarity of our methods, the transparency of our reporting, and the cautious interpretation of our findings. We have addressed each comment point by point below.

**REVIEWER #1 COMMENTS**

**Comment R1.1.** *“The comparison ‘synbiotics plus iron vs. standard control with or without iron’ pools two fundamentally different comparators: one where the control arm receives iron and one where it does not. The clinical heterogeneity is not adequately addressed. The footnote acknowledges this but the justification for pooling is missing. A sensitivity analysis excluding the study with a non-iron comparator could be considered and the reasons why the pooled estimate remained informative could be discussed.”*

**Response:** We thank the Reviewer for this observation. The comparison of synbiotics plus iron includes only two RCTs: Helmyati 2020, in which the comparator arm received iron, and Lovell 2018, in which the comparator was a placebo without iron. We re-examined both trials individually and found that their results were consistent: both reported a small increase in hemoglobin in the intervention arm (+0.60 and +0.59 g/dL, respectively), well below our predefined minimal important difference (MID) of 1.0 g/dL, and the pooled estimate (MD: +0.23 g/dL; 95% CI: −0.09 to +0.56) did not reach or cross the MID. The statistical heterogeneity was low. Because excluding the trial with the non-iron comparator (Lovell 2018) would leave the estimate essentially unchanged and would not modify the interpretation (no clinically important effect on hemoglobin, very low certainty), we corroborated that the results were consistent and considered that a formal sensitivity analysis was not necessary, as it would not alter the interpretation. We have added a brief clarifying sentence to the Results section to make this explicit.

***Modified text in the manuscript (Results):*** *“Compared with the control, with or without iron, symbiotics with iron may not importantly increase hemoglobin (2 RCTs; MD: +0.23 g/dL; 95% CI: -0.09 to +0.56; very low certainty). Results were similar regardless of whether the comparator included iron.”*

**Comment R1.2.** *“Several outcomes are based on only one RCT, yet conclusions have been drawn from them: • Anemia with probiotics vs. placebo – 1 RCT (in table 3 and in the discussion & conclusion) • Hematocrit with probiotics vs. placebo – 1 RCT (in table 3 and results section) • Anemia with synbiotics + iron – 1 RCT (in table 6 and results section). The single-study findings are presented in the same format as meta-analysis findings and carried forward into the abstract & conclusion as a noteworthy result. However, they should be clearly distinguished as single-study findings from pooled estimates or preliminary, single-trial evidence.”*

**Response:** We appreciate this comment and would like to clarify our analytical framework. The outcomes informed by a single trial are not presented as pooled meta-analytic estimates; they are reported within the GRADE Summary of Findings (SoF) tables because a single RCT still constitutes the available body of evidence for that outcome, and the number of contributing studies is explicitly indicated (e.g., ‘1 RCT’). Under the minimally contextualized GRADE approach, the certainty of the evidence does not depend on whether an estimate derives from one or several studies, but on the certainty domains, particularly imprecision [21]. A single, typically small trial yields wide 95% confidence intervals and is downgraded for imprecision, which is precisely why these outcomes (anemia and hematocrit with probiotics vs. placebo; anemia with synbiotics plus iron) are rated as low or very low certainty. The interpretation therefore rests on where the point estimate and its confidence interval fall relative to the MID and on the certainty rating, not on the number of studies.

**Comment R1.3.** *“The included studies heterogeneous pediatric age range from 0.5 years to ~11 years. No subgroup or sensitivity analysis was performed by age group. Given that iron physiology, gut microbiome composition, and intervention responsiveness differ substantially across early infancy, childhood, and adolescence, this is an important limitation. This has been mentioned in the discussion, but not adequately addressed why subgroup analysis was not performed.”*

**Response:** As stated in the Methods, subgroup analyses were not performed because, in general, there was no statistical heterogeneity across the outcomes. In addition, the number of studies per comparison was small (2–5), which makes formal subgroup analysis or meta-regression by age underpowered and prone to spurious findings. The absence of statistical heterogeneity, together with consistent point estimates across trials that spanned different pediatric age ranges, suggests that the (predominantly null) effects were consistent across age groups. We have clarified this rationale in the Methods.

***Modified text in the manuscript (Discussion):*** *“Notably, the consistency of the point estimates across trials spanning different age ranges, together with the absence of statistical heterogeneity, suggests that age did not meaningfully modify the efficacy of the interventions.”*

**Comment R1.4.** *“Table 3 is titled ‘Summary of results of the effects of prebiotics versus placebo’, but it clearly reports probiotics data. This must be corrected.”*

**Response:** We thank the Reviewer for detecting this error. The title of Table 3 has been corrected from ‘prebiotics versus placebo’ to ‘probiotics versus placebo’.

**REVIEWER #2 COMMENTS**

**Comment R2.1.** *“The included studies involve highly heterogeneous interventions (different strains, substrates, dosing, duration, and co-interventions), yet are pooled broadly. Probiotics include multiple strains (e.g., Lactobacillus spp.), while prebiotics also vary (GOS, FOS, inulin). Pooling such heterogeneous interventions may obscure true effects and reduce interpretability.”*

**Response:** We agree that the interventions differ in strains, substrates (e.g., GOS, FOS, inulin), doses, and durations. However, despite this clinical heterogeneity, the results were consistent in showing no clinically important effect on the evaluated outcomes across comparisons, and the statistical heterogeneity (I²) was low in the analyses. As addressed in the Discussion, we predefined six comparisons grouped by intervention type (prebiotics, probiotics, and synbiotics, each with or without iron) precisely to obtain more concrete and interpretable questions. Because the statistical heterogeneity was low, subgroup or sensitivity analyses by strain, substrate, dose, or duration were not warranted and would not change the interpretation. We nonetheless acknowledge, as already noted in the Discussion, that this consistency does not exclude biological differences between specific strains or compounds.

**Comment R2.2.** *“Differentiate statistical significance and clinical relevance (based on MID). The anemia reduction with probiotics is based on a single RCT with low certainty.”*

**Response:** We would like to emphasize that our analysis did not interpret the results according to statistical significance. Under the minimally contextualized GRADE approach, results are interpreted according to where the point estimate and its 95% confidence interval fall relative to the predefined MID, together with the certainty of the evidence — not according to p-values or statistical significance. Consistent with this, the reduction in anemia with probiotics is based on a single RCT, is explicitly rated as low certainty, and has a 95% confidence interval that crosses the MID; it is therefore not interpreted as a definitive or clinically important effect.

**Comment R2.3.** *“The review appropriately assesses risk of bias using Cochrane RoB and GRADE. Many included studies are, however, at high/unclear risk due to incomplete outcome data and selective reporting. Despite this, some outcomes are rated as high certainty. Justify how high-risk studies influenced pooled estimates. Consider presenting sensitivity analysis excluding high-risk studies.”*

**Response:** We appreciate this observation. In the GRADE framework, risk of bias is addressed by downgrading the certainty of the evidence based on the qualitative assessment of the included studies and their relative weight within each meta-analysis. Outcomes that retained high certainty were those in which the studies at high risk of bias contributed little weight so that downgrading was not warranted. Importantly, we found no statistical heterogeneity to suggest that the estimates differed systematically between studies at low and high risk of bias; consequently, a sensitivity analysis excluding high-risk studies would be unlikely to change the pooled estimates or the conclusions, and we did not consider it necessary.

**Comment R2.4.** *“Review the manuscript and correct errors. Symbiotics is used in parts that look like it should be synbiotics. The eligibility criteria for example lists symbiotics but not synbiotics, is this correct?”*

**Response:** We thank the Reviewer for noting this inconsistency. The correct term for the combination of probiotics and prebiotics is ‘synbiotics’. We have replaced ‘symbiotics’ with ‘synbiotics’ consistently throughout the manuscript.
