# Supplementary material for: Efficacy and safety of prebiotics, probiotics, and synbiotics on hemoglobin and anemia in the pediatric population: A systematic review and meta-analysis
Source: PLoS One. 2026 Jul 29;21(7):e0354681. doi: 10.1371/journal.pone.0354681 (PMC13419176; doi:10.1371/journal.pone.0354681)
Supplement: Peer review history

# Peer review: Decision Letter 0

**DOI:** 10.1371/journal.pone.0354681.r001

**Authors:** Fahrul Nurkolis

23 Jun 2026

PONE-D-26-15986

Efficacy and safety of prebiotics, probiotics, and symbiotics on hemoglobin and anemia in the pediatric population: A systematic review and meta-Analysis

PLOS One

Dear Dr. Soriano-Moreno,

Thank you for submitting your manuscript to PLOS ONE. After careful consideration, we feel that it has merit but does not fully meet PLOS ONE’s publication criteria as it currently stands. Therefore, we invite you to submit a revised version of the manuscript that addresses the points raised during the review process.

Please submit your revised manuscript by Aug 08 2026 11:59PM. If you will need more time than this to complete your revisions, please reply to this message or contact the journal office at plosone@plos.org. When you're ready to submit your revision, log on to https://www.editorialmanager.com/pone/ and select the 'Submissions Needing Revision' folder to locate your manuscript file.

Please include the following items when submitting your revised manuscript:

- A letter that responds to each point raised by the academic editor and reviewer(s). You should upload this letter as a separate file labeled 'Response to Reviewers'.
- A marked-up copy of your manuscript that highlights changes made to the original version. You should upload this as a separate file labeled 'Revised Manuscript with Track Changes'.
- An unmarked version of your revised paper without tracked changes. You should upload this as a separate file labeled 'Manuscript'.

If you would like to make changes to your financial disclosure, please include your updated statement in your cover letter. Guidelines for resubmitting your figure files are available below the reviewer comments at the end of this letter.

If applicable, we recommend that you deposit your laboratory protocols in protocols.io to enhance the reproducibility of your results. Protocols.io assigns your protocol its own identifier (DOI) so that it can be cited independently in the future. For instructions see: https://journals.plos.org/plosone/s/submission-guidelines#loc-laboratory-protocols. Additionally, PLOS ONE offers an option for publishing peer-reviewed Lab Protocol articles, which describe protocols hosted on protocols.io. Read more information on sharing protocols at https://plos.org/protocols?utm_medium=editorial-email&utm_source=authorletters&utm_campaign=protocols.

As the corresponding author, your ORCID iD is verified in the submission system and will appear in the published article. PLOS supports the use of ORCID, and we encourage all coauthors to register for an ORCID iD and use it as well. Please encourage your coauthors to verify their ORCID iD within the submission system before final acceptance, as unverified ORCID iDs will not appear in the published article. Only the individual author can complete the verification step; PLOS staff cannot verify ORCID iDs on behalf of authors.

We look forward to receiving your revised manuscript.

Kind regards,

Prof. (H.C.) Fahrul Nurkolis, B.Sc., M.Biomed

Academic Editor

PLOS One

Journal Requirements:

When submitting your revision, we need you to address these additional requirements.

1. Please ensure that your manuscript meets PLOS ONE's style requirements, including those for file naming. The PLOS ONE style templates can be found at

https://journals.plos.org/plosone/s/file?id=wjVg/PLOSOne_formatting_sample_main_body.pdf and

https://journals.plos.org/plosone/s/file?id=ba62/PLOSOne_formatting_sample_title_authors_affiliations.pdf

2. Please ensure that you have uploaded your PRISMA flowchart as Figure 1, or else explain why this is not possible. Blank flowcharts can be found here: http://www.prisma-statement.org/

3. If the reviewer comments include a recommendation to cite specific previously published works, please review and evaluate these publications to determine whether they are relevant and should be cited. There is no requirement to cite these works unless the editor has indicated otherwise.

[Note: HTML markup is below. Please do not edit.]

Reviewers' comments:

Reviewer's Responses to Questions

**Comments to the Author**

1. Is the manuscript technically sound, and do the data support the conclusions?

The manuscript must describe a technically sound piece of scientific research with data that supports the conclusions. Experiments must have been conducted rigorously, with appropriate controls, replication, and sample sizes. The conclusions must be drawn appropriately based on the data presented.

Reviewer #1: No

Reviewer #2: Yes

**********

2. Has the statistical analysis been performed appropriately and rigorously?

Reviewer #1: Yes

Reviewer #2: Yes

**********

3. Have the authors made all data underlying the findings in their manuscript fully available?

The PLOS Data policy requires authors to make all data underlying the findings described in their manuscript fully available without restriction, with rare exception (please refer to the Data Availability Statement in the manuscript PDF file). The data should be provided as part of the manuscript or its supporting information, or deposited to a public repository. For example, in addition to summary statistics, the data points behind means, medians and variance measures should be available. If there are restrictions on publicly sharing data—e.g. participant privacy or use of data from a third party—those must be specified.

Reviewer #1: Yes

Reviewer #2: Yes

**********

4. Is the manuscript presented in an intelligible fashion and written in standard English?

PLOS ONE does not copyedit accepted manuscripts, so the language in submitted articles must be clear, correct, and unambiguous. Any typographical or grammatical errors should be corrected at revision, so please note any specific errors here.

Reviewer #1: Yes

Reviewer #2: Yes

**********

5. Review Comments to the Author

Please use the space provided to explain your answers to the questions above. You may also include additional comments for the author, including concerns about dual publication, research ethics, or publication ethics. (Please upload your review as an attachment if it exceeds 20,000 characters)

Reviewer #1: The authors have followed established methodological standards, including PRISMA, GRADE, and PROSPERO registration, and have clearly communicated the findings.

Instead of focusing only on statistical significance, the authors defined Minimal Important Differences (MIDs) for each outcome in advance and used these to interpret the findings. This is a thoughtful and rigorous approach that strengthens the interpretation of the results.

A few comments:

1. The comparison ‘synbiotics plus iron vs. standard control with or without iron’ pools two fundamentally different comparators: one where the control arm receives iron and one where it does not. The clinical heterogeneity is not adequately addressed. The footnote acknowledges this but the justification for pooling is missing.

A sensitivity analysis excluding the study with a non-iron comparator could be considered and the reasons why the pooled estimate remained informative could be discussed.

2. Several outcomes are based on only one RCT, yet conclusions have been drawn from them:

• Anemia with probiotics vs. placebo- 1 RCT (in table 3 and in the discussion & conclusion)

• Hematocrit with probiotics vs. placebo- 1 RCT (in table 3 and results section)

• Anemia with synbiotics + iron-1 RCT (in table 6 and results section)

The single-study findings are presented in the same format as meta-analysis findings and carried forward into the abstract & conclusion as a noteworthy result. However, they should be clearly distinguished as single-study findings from pooled estimates or preliminary, single-trial evidence.

3. The included studies heterogenous pediatric age range from 0.5 years to ~11 years. No subgroup or sensitivity analysis was performed by age group. Given that iron physiology, gut microbiome composition, and intervention responsiveness differ substantially across early infancy, childhood, and adolescence, this is an important limitation. This has been mentioned in the discussion, but not adequately addressed why subgroup analysis was not performed.

4. Table 3 is titled ‘Summary of results of the effects of prebiotics versus placebo’, but it clearly reports probiotics data. This must be corrected.

Reviewer #2: The systematic review is well conducted and addresses an important and policy-relevant question about microbiome-based interventions for pediatric anemia.

The manuscript claims to improve on previous reviews by separating intervention types and applying GRADE with MID thresholds. While this is valuable, the added contribution is not sufficiently demonstrated

The included studies involve highly heterogeneous interventions (different strains, substrates, dosing, duration, and co-interventions), yet are pooled broadly. Probiotics include multiple strains (e.g., Lactobacillus spp.), while prebiotics also vary (GOS, FOS, inulin). Pooling such heterogeneous interventions may obscure true effects and reduce interpretability.

Differentiate statistical significance and clinical relevance (based on MID). The anemia reduction with probiotics is based on a single RCT with low certainty.

The review appropriately assesses risk of bias using Cochrane RoB and GRADE. Many included studies are, however, at high/unclear risk due to incomplete outcome data and selective reporting. Despite this, some outcomes are rated as high certainty. Justify how high-risk studies influenced pooled estimates. Consider presenting sensitivity analysis excluding high-risk studies.

Review the manuscript and correct errors. Symbiotics is used in parts that look like it should be synbiotics. The eligibility criteria for example lists symbiotics but not synbiotics, is this correct?

**********

6. PLOS authors have the option to publish the peer review history of their article (what does this mean?). If published, this will include your full peer review and any attached files.

If you choose “no”, your identity will remain anonymous but your review may still be made public.

**Do you want your identity to be public for this peer review?**  For information about this choice, including consent withdrawal, please see our Privacy Policy.

Reviewer #1: **Yes:**  Anju Pradhan Sinha

Reviewer #2: No

**********

[NOTE: If reviewer comments were submitted as an attachment file, they will be attached to this email and accessible via the submission site. Please log into your account, locate the manuscript record, and check for the action link "View Attachments". If this link does not appear, there are no attachment files.]

To ensure your figures meet our technical requirements, please review our figure guidelines: https://journals.plos.org/plosone/s/figures

You may also use PLOS’s free figure tool, NAAS, to help you prepare publication quality figures: https://journals.plos.org/plosone/s/figures#loc-tools-for-figure-preparation.

NAAS will assess whether your figures meet our technical requirements by comparing each figure against our figure specifications.

Attachment Submitted filename: Review_PLOS One manuscript.docx

# Author comment: Author response to Decision Letter 1

**DOI:** 10.1371/journal.pone.0354681.r002

1 Jul 2026

RESPONSE TO REVIEWERS

Manuscript title: Efficacy and safety of prebiotics, probiotics, and synbiotics on hemoglobin and anemia in the pediatric population: A systematic review and meta-analysis

Manuscript ID: PONE-D-26-15986

We sincerely thank both reviewers for their careful, rigorous, and constructive evaluation of our manuscript. Their comments have helped us improve the clarity of our methods, the transparency of our reporting, and the cautious interpretation of our findings. We have addressed each comment point by point below.

REVIEWER #1 COMMENTS

Comment R1.1. “The comparison ‘synbiotics plus iron vs. standard control with or without iron’ pools two fundamentally different comparators: one where the control arm receives iron and one where it does not. The clinical heterogeneity is not adequately addressed. The footnote acknowledges this but the justification for pooling is missing. A sensitivity analysis excluding the study with a non-iron comparator could be considered and the reasons why the pooled estimate remained informative could be discussed.”

Response: We thank the Reviewer for this observation. The comparison of synbiotics plus iron includes only two RCTs: Helmyati 2020, in which the comparator arm received iron, and Lovell 2018, in which the comparator was a placebo without iron. We re-examined both trials individually and found that their results were consistent: both reported a small increase in hemoglobin in the intervention arm (+0.60 and +0.59 g/dL, respectively), well below our predefined minimal important difference (MID) of 1.0 g/dL, and the pooled estimate (MD: +0.23 g/dL; 95% CI: −0.09 to +0.56) did not reach or cross the MID. The statistical heterogeneity was low. Because excluding the trial with the non-iron comparator (Lovell 2018) would leave the estimate essentially unchanged and would not modify the interpretation (no clinically important effect on hemoglobin, very low certainty), we corroborated that the results were consistent and considered that a formal sensitivity analysis was not necessary, as it would not alter the interpretation. We have added a brief clarifying sentence to the Results section to make this explicit.

Modified text in the manuscript (Results): “Compared with the control, with or without iron, symbiotics with iron may not importantly increase hemoglobin (2 RCTs; MD: +0.23 g/dL; 95% CI: -0.09 to +0.56; very low certainty). Results were similar regardless of whether the comparator included iron.”

Comment R1.2. “Several outcomes are based on only one RCT, yet conclusions have been drawn from them: • Anemia with probiotics vs. placebo – 1 RCT (in table 3 and in the discussion & conclusion) • Hematocrit with probiotics vs. placebo – 1 RCT (in table 3 and results section) • Anemia with synbiotics + iron – 1 RCT (in table 6 and results section). The single-study findings are presented in the same format as meta-analysis findings and carried forward into the abstract & conclusion as a noteworthy result. However, they should be clearly distinguished as single-study findings from pooled estimates or preliminary, single-trial evidence.”

Response: We appreciate this comment and would like to clarify our analytical framework. The outcomes informed by a single trial are not presented as pooled meta-analytic estimates; they are reported within the GRADE Summary of Findings (SoF) tables because a single RCT still constitutes the available body of evidence for that outcome, and the number of contributing studies is explicitly indicated (e.g., ‘1 RCT’). Under the minimally contextualized GRADE approach, the certainty of the evidence does not depend on whether an estimate derives from one or several studies, but on the certainty domains, particularly imprecision [21]. A single, typically small trial yields wide 95% confidence intervals and is downgraded for imprecision, which is precisely why these outcomes (anemia and hematocrit with probiotics vs. placebo; anemia with synbiotics plus iron) are rated as low or very low certainty. The interpretation therefore rests on where the point estimate and its confidence interval fall relative to the MID and on the certainty rating, not on the number of studies.

Comment R1.3. “The included studies heterogeneous pediatric age range from 0.5 years to ~11 years. No subgroup or sensitivity analysis was performed by age group. Given that iron physiology, gut microbiome composition, and intervention responsiveness differ substantially across early infancy, childhood, and adolescence, this is an important limitation. This has been mentioned in the discussion, but not adequately addressed why subgroup analysis was not performed.”

Response: As stated in the Methods, subgroup analyses were not performed because, in general, there was no statistical heterogeneity across the outcomes. In addition, the number of studies per comparison was small (2–5), which makes formal subgroup analysis or meta-regression by age underpowered and prone to spurious findings. The absence of statistical heterogeneity, together with consistent point estimates across trials that spanned different pediatric age ranges, suggests that the (predominantly null) effects were consistent across age groups. We have clarified this rationale in the Methods.

Modified text in the manuscript (Discussion): “Notably, the consistency of the point estimates across trials spanning different age ranges, together with the absence of statistical heterogeneity, suggests that age did not meaningfully modify the efficacy of the interventions.”

Comment R1.4. “Table 3 is titled ‘Summary of results of the effects of prebiotics versus placebo’, but it clearly reports probiotics data. This must be corrected.”

Response: We thank the Reviewer for detecting this error. The title of Table 3 has been corrected from ‘prebiotics versus placebo’ to ‘probiotics versus placebo’.

REVIEWER #2 COMMENTS

Comment R2.1. “The included studies involve highly heterogeneous interventions (different strains, substrates, dosing, duration, and co-interventions), yet are pooled broadly. Probiotics include multiple strains (e.g., Lactobacillus spp.), while prebiotics also vary (GOS, FOS, inulin). Pooling such heterogeneous interventions may obscure true effects and reduce interpretability.”

Response: We agree that the interventions differ in strains, substrates (e.g., GOS, FOS, inulin), doses, and durations. However, despite this clinical heterogeneity, the results were consistent in showing no clinically important effect on the evaluated outcomes across comparisons, and the statistical heterogeneity (I²) was low in the analyses. As addressed in the Discussion, we predefined six comparisons grouped by intervention type (prebiotics, probiotics, and synbiotics, each with or without iron) precisely to obtain more concrete and interpretable questions. Because the statistical heterogeneity was low, subgroup or sensitivity analyses by strain, substrate, dose, or duration were not warranted and would not change the interpretation. We nonetheless acknowledge, as already noted in the Discussion, that this consistency does not exclude biological differences between specific strains or compounds.

Comment R2.2. “Differentiate statistical significance and clinical relevance (based on MID). The anemia reduction with probiotics is based on a single RCT with low certainty.”

Response: We would like to emphasize that our analysis did not interpret the results according to statistical significance. Under the minimally contextualized GRADE approach, results are interpreted according to where the point estimate and its 95% confidence interval fall relative to the predefined MID, together with the certainty of the evidence — not according to p-values or statistical significance. Consistent with this, the reduction in anemia with probiotics is based on a single RCT, is explicitly rated as low certainty, and has a 95% confidence interval that crosses the MID; it is therefore not interpreted as a definitive or clinically important effect.

Comment R2.3. “The review appropriately assesses risk of bias using Cochrane RoB and GRADE. Many included studies are, however, at high/unclear risk due to incomplete outcome data and selective reporting. Despite this, some outcomes are rated as high certainty. Justify how high-risk studies influenced pooled estimates. Consider presenting sensitivity analysis excluding high-risk studies.”

Response: We appreciate this observation. In the GRADE framework, risk of bias is addressed by downgrading the certainty of the evidence based on the qualitative assessment of the included studies and their relative weight within each meta-analysis. Outcomes that retained high certainty were those in which the studies at high risk of bias contributed little weight so that downgrading was not warranted. Importantly, we found no statistical heterogeneity to suggest that the estimates differed systematically between studies at low and high risk of bias; consequently, a sensitivity analysis excluding high-risk studies would be unlikely to change the pooled estimates or the conclusions, and we did not consider it necessary.

Comment R2.4. “Review the manuscript and correct errors. Symbiotics is used in parts that look like it should be synbiotics. The eligibility criteria for example lists symbiotics but not synbiotics, is this correct?”

Response: We thank the Reviewer for noting this inconsistency. The correct term for the combination of probiotics and prebiotics is ‘synbiotics’. We have replaced ‘symbiotics’ with ‘synbiotics’ consistently throughout the manuscript.

Attachment Submitted filename: Response to reviewers - COMPLETED.docx

# Peer review: Decision Letter 1

**DOI:** 10.1371/journal.pone.0354681.r003

**Authors:** Fahrul Nurkolis

12 Jul 2026

Efficacy and safety of prebiotics, probiotics, and symbiotics on hemoglobin and anemia in the pediatric population: A systematic review and meta-Analysis

PONE-D-26-15986R1

Dear Dr. Soriano-Moreno,

We’re pleased to inform you that your manuscript has been judged scientifically suitable for publication and will be formally accepted for publication once it meets all outstanding technical requirements.

Within one week, you’ll receive an e-mail detailing the required amendments. When these have been addressed, you’ll receive a formal acceptance letter and your manuscript will be scheduled for publication.

An invoice will be generated when your article is formally accepted. Please note, if your institution has a publishing partnership with PLOS and your article meets the relevant criteria, all or part of your publication costs will be covered. Please make sure your user information is up-to-date by logging into Editorial Manager at Editorial Manager® and clicking the ‘Update My Information' link at the top of the page. For questions related to billing, please contact billing support.

If your institution or institutions have a press office, please notify them about your upcoming paper to help maximize its impact. If they’ll be preparing press materials, please inform our press team as soon as possible -- no later than 48 hours after receiving the formal acceptance. Your manuscript will remain under strict press embargo until 2 pm Eastern Time on the date of publication. For more information, please contact onepress@plos.org.

Kind regards,

Fahrul Nurkolis

Academic Editor

PLOS One

Additional Editor Comments (optional):

Reviewers' comments:

Reviewer's Responses to Questions

**Comments to the Author**

1. If the authors have adequately addressed your comments raised in a previous round of review and you feel that this manuscript is now acceptable for publication, you may indicate that here to bypass the “Comments to the Author” section, enter your conflict of interest statement in the “Confidential to Editor” section, and submit your "Accept" recommendation.

Reviewer #2: (No Response)

**********

2. Is the manuscript technically sound, and do the data support the conclusions?

The manuscript must describe a technically sound piece of scientific research with data that supports the conclusions. Experiments must have been conducted rigorously, with appropriate controls, replication, and sample sizes. The conclusions must be drawn appropriately based on the data presented.

Reviewer #2: (No Response)

**********

3. Has the statistical analysis been performed appropriately and rigorously?

Reviewer #2: (No Response)

**********

4. Have the authors made all data underlying the findings in their manuscript fully available?

The PLOS Data policy requires authors to make all data underlying the findings described in their manuscript fully available without restriction, with rare exception (please refer to the Data Availability Statement in the manuscript PDF file). The data should be provided as part of the manuscript or its supporting information, or deposited to a public repository. For example, in addition to summary statistics, the data points behind means, medians and variance measures should be available. If there are restrictions on publicly sharing data—e.g. participant privacy or use of data from a third party—those must be specified.

Reviewer #2: (No Response)

**********

5. Is the manuscript presented in an intelligible fashion and written in standard English?

PLOS ONE does not copyedit accepted manuscripts, so the language in submitted articles must be clear, correct, and unambiguous. Any typographical or grammatical errors should be corrected at revision, so please note any specific errors here.

Reviewer #2: (No Response)

**********

6. Review Comments to the Author

Please use the space provided to explain your answers to the questions above. You may also include additional comments for the author, including concerns about dual publication, research ethics, or publication ethics. (Please upload your review as an attachment if it exceeds 20,000 characters)

Reviewer #2: The authors adequately addressed my comments, which were related to microorganism strains, substrates, dosing, duration, and co-interventions; clinical relevance; and the proper use of the term synbiotics.

**********

7. PLOS authors have the option to publish the peer review history of their article (what does this mean?). If published, this will include your full peer review and any attached files.

If you choose “no”, your identity will remain anonymous but your review may still be made public.

**Do you want your identity to be public for this peer review?**  For information about this choice, including consent withdrawal, please see our Privacy Policy.

Reviewer #2: No

**********

# Editor report: Acceptance letter

**DOI:** 10.1371/journal.pone.0354681.r004

**Authors:** Fahrul Nurkolis

PONE-D-26-15986R1

PLOS One

Dear Dr. Soriano-Moreno,

I'm pleased to inform you that your manuscript has been deemed suitable for publication in PLOS One. Congratulations! Your manuscript is now being handed over to our production team.

At this stage, our production department will prepare your paper for publication. This includes ensuring the following:

* All references, tables, and figures are properly cited

* All relevant supporting information is included in the manuscript submission,

* There are no issues that prevent the paper from being properly typeset

You will receive further instructions from the production team, including instructions on how to review your proof when it is ready. Please keep in mind that we are working through a large volume of accepted articles, so please give us a few days to review your paper and let you know the next and final steps.

Lastly, if your institution or institutions have a press office, please let them know about your upcoming paper now to help maximize its impact. If they'll be preparing press materials, please inform our press team within the next 48 hours. Your manuscript will remain under strict press embargo until 2 pm Eastern Time on the date of publication. For more information, please contact onepress@plos.org.

You will receive an invoice from PLOS for your publication fee after your manuscript has reached the completed accept phase. If you receive an email requesting payment before acceptance or for any other service, this may be a phishing scheme. Learn how to identify phishing emails and protect your accounts at https://explore.plos.org/phishing.

If we can help with anything else, please email us at customercare@plos.org.

Thank you for submitting your work to PLOS ONE and supporting open access.

Kind regards,

PLOS ONE Editorial Office Staff

on behalf of

Dr. Fahrul Nurkolis

Academic Editor

PLOS One
